# Partitioned polygenic risk scores identify distinct types of metabolic dysfunction-associated steatotic liver disease

**DOI:** 10.1038/s41591-024-03284-0

**Published:** 2024-12-09

**Authors:** Oveis Jamialahmadi, Antonio De Vincentis, Federica Tavaglione, Francesco Malvestiti, Ruifang Li-Gao, Rosellina M. Mancina, Marcus Alvarez, Kyla Gelev, Samantha Maurotti, Umberto Vespasiani-Gentilucci, Frits Richard Rosendaal, Julia Kozlitina, Päivi Pajukanta, François Pattou, Luca Valenti, Stefano Romeo

**Affiliations:** 1 Department of Molecular and Clinical Medicine, Institute of Medicine, Sahlgrenska Academy, Wallenberg Laboratory, University of Gothenburg, Gothenburg, Sweden; 2 Operative Unit of Internal Medicine, Fondazione Policlinico Universitario Campus Bio-Medico, Rome, Italy; 3 Research Unit of Internal Medicine, Department of Medicine and Surgery, Università Campus Bio-Medico di Roma, Rome, Italy; 4 Operative Unit of Clinical Medicine and Hepatology, Fondazione Policlinico Universitario Campus Bio-Medico, Rome, Italy; 5 Research Unit of Clinical Medicine and Hepatology, Department of Medicine and Surgery, Università Campus Bio-Medico di Roma, Rome, Italy; 6 Department of Pathophysiology and Transplantation, Università degli Studi di Milano, Milan, Italy; 7 Department of Clinical Epidemiology, Leiden University Medical Center, Leiden, the Netherlands; 8 Department of Life Science, Health, and Health Professions, Link Campus University, Rome, Italy; 9 Department of Human Genetics, David Geffen School of Medicine at UCLA, Los Angeles, CA USA; 10 Department of Experimental and Clinical Medicine, Magna Graecia University, Catanzaro, Italy; 11 The Eugene McDermott Center for Human Growth and Development, University of Texas Southwestern Medical Center, Dallas, TX USA; 12 Bioinformatics Interdepartmental Program, UCLA, Los Angeles, CA USA; 13 Institute for Precision Health, David Geffen School of Medicine at UCLA, Los Angeles, CA USA; 14 Service de chirurgie générale et endocrinienne, Centre Hospitalier Universitaire de Lille, Lille, France; 15 European Genomic Institute for Diabetes, UMR 1190 Translational Research for Diabetes, Inserm, CHU Lille, University of Lille, Lille, France; 16 Precision Medicine - Biological Resource Center, Department of Transfusion Medicine, Fondazione IRCCS Ca’ Granda Ospedale Maggiore Policlinico, Milan, Italy; 17 Department of Cardiology, Sahlgrenska University Hospital, Gothenburg, Sweden; 18 Clinical Nutrition Unit, Department of Medical and Surgical Sciences, University Magna Graecia, Catanzaro, Italy; 19 Department of Medicine (H7), Karolinska Institute, Huddinge, Stockholm, Sweden; 20 Department of Endocrinology, Karolinska University Hospital, Huddinge, Stockholm, Sweden

**Keywords:** Risk factors, Genome-wide association studies

## Abstract

Metabolic dysfunction-associated steatotic liver disease (MASLD) is characterized by an excess of lipids, mainly triglycerides, in the liver and components of the metabolic syndrome, which can lead to cirrhosis and liver cancer. While there is solid epidemiological evidence that MASLD clusters with cardiometabolic disease, several leading genetic risk factors for MASLD do not increase the risk of cardiovascular disease, suggesting no causal relationship between MASLD and cardiometabolic derangement. In this work, we leveraged measurements of visceral adiposity identifying 27 previously unknown genetic loci associated with MASLD (*n* = 36,394), six replicated in four independent cohorts (*n* = 3,903). Next, we generated two partitioned polygenic risk scores based on the presence of lipoprotein retention in the liver. The two polygenic risk scores suggest the presence of at least two distinct types of MASLD, one confined to the liver resulting in a more aggressive liver disease and one that is systemic and results in a higher risk of cardiometabolic disease. These findings shed light on the heterogeneity of MASLD and have the potential to improve the prediction of clinical trajectories and inform precision medicine approaches.

## Abstract (web summary)

The application of partitioned polygenic scores to data from cohorts of patients identifies two distinct subtypes of MASLD with different cardiometabolic risk profiles.

## Main

Paralleling the obesity epidemic, steatotic liver disease (SLD) is a growing burden worldwide. SLD includes a spectrum of conditions characterized by an excess of lipids, mainly triglycerides, stored in intracellular lipid droplets in the liver, potentially progressing to inflammation, fibrosis and ultimately to cirrhosis and liver cancer^1^. SLD is a heterogenous disease coexisting with a metabolic derangement, including visceral adiposity, insulin resistance and hypertension, namely, metabolic dysfunction-associated SLD (or MASLD). This metabolic derangement ultimately increases the risk of cardiovascular events, including heart failure, and also increases kidney disease^2–4^. Indeed, cardiovascular disease is the most frequent cause of death in individuals with MASLD, whereas liver-related death is less frequent; however, it is a common clinical observation that some individuals develop a rapidly progressing liver disease despite similar or even less-marked metabolic derangement.

MASLD has a strong inherited component; several variants that increase primarily liver lipids by impairing hepatocyte lipid droplet remodeling and lipoprotein secretion also cause the progression of MASLD^5^; however, contrarily to the epidemiological evidence, these variants result in a protection against cardiovascular disease and no association with hypertension^5–7^ or heart failure, suggesting no causal relationship between MASLD and cardiometabolic derangement^5^.

Over the last 15 years, genome-wide association studies (GWAS) identified several genetic loci associated with chronic liver disease or proxies for increased liver triglyceride content^8–13^. Excess in adiposity amplifies the effect size of a handful of variants^14^ likely by increasing ectopic visceral fat. To improve the precision of genetic studies and to identify genetic variants with primary effects on the liver, independent of adiposity, GWAS analyses are typically adjusted for body mass index (BMI); however, anthropometric measures of adiposity (BMI) and body fat distribution (waist circumference) fail to provide an accurate quantification of visceral adiposity, which is most closely related to insulin resistance and metabolic alterations. Therefore, standard adjustments for BMI may fail to capture and remove the total effect of adiposity on liver fat, limiting the precision of GWAS. In contrast, imaging (for example, visceral adipose volume) and bioelectrical impedance analysis (for example, whole-body fat mass) are more accurate measurements of body composition and are better predictors of MASLD^15^. We thus reasoned that adjusting for these traits could better capture the effect of adiposity on liver fat, thereby improving the power to detect previously unknown loci contributing to SLD.

Here, we show that indices of adiposity differentially contribute to the association between genetic variants and liver triglyceride content/inflammation, and we leverage these indices to identify previously unknown genetic loci associated with SLD. We identified and replicated six previously unknown loci and generated two partitioned polygenic risk scores (pPRSs) that suggest the presence of at least two distinct types of MASLD, one confined to the liver and one entwined in the systemic cardiometabolic syndrome.

## Results

### Visceral adipose tissue, whole-body fat mass and BMI are independent predictors of liver triglyceride content and inflammation/fibrosis

**Extended Data Fig. 1:**
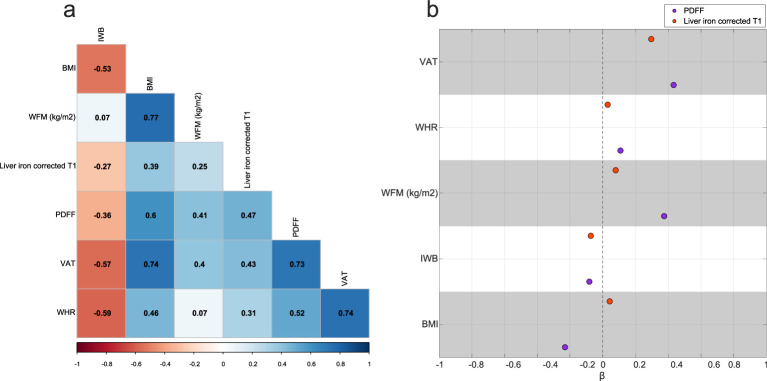
Measures of adiposity are highly correlated with liver triglycerides and inflammation/fibrosis, with VAT, WFM, and BMI being independent predictors of liver outcomes. In (**a**), the phenotypic correlation between different measures of adiposity, liver triglyceride content measured by proton density fat fraction (PDFF), and inflammation/fibrosis measured by liver iron corrected T1 (cT1); pairwise Spearman’s correlation coefficients have been shown on the heatmap. All correlations had a Benjamini–Hochberg False Discovery Rate (FDR) <0.05. (**b**) penalized Ridge regression analysis of different adiposity indices in predicting PDFF and liver iron corrected T1. Each dot represents standardized coefficients, and dashed line represents the lack of contribution of each trait to the liver outcomes. Both target variables were rank-based inverse normal transformed before the regression analysis. VAT: visceral adipose tissue, WFM: whole-body fat mass, BMI: body mass index, IWB: impedance of whole body, WHR: waist-to-hip ratio.

To identify the independent predictors of liver triglyceride content and inflammation/fibrosis among the indices of adiposity, we examined the pairwise correlations among different measures of adiposity and (1) liver triglyceride content measured by magnetic resonance imaging (MRI)-derived proton density fat fraction (PDFF); and (2) liver inflammation/fibrosis measured by liver iron corrected T1 (cT1) in European participants from the UK Biobank (Extended Data Fig. 1a). The strongest correlation with liver traits was observed for visceral adipose tissue (VAT) volume followed by BMI, waist-to-hip ratio (WHR) and whole-body fat mass (WFM). As expected, there was a high correlation between PDFF and cT1. Due to high multicollinearity among the adiposity indices, we used three penalized regression models (Methods) to assess their predictive contribution to PDFF and cT1. The standardized coefficients from the best performing algorithm (Ridge regression) showed that VAT was the strongest independent predictor of PDFF and cT1, followed by WFM and BMI for PDFF (Extended Data Fig. 1b). In the penalized regression analysis, WHR and impedance of whole body had almost no independent predictive power and therefore, we used WFM, BMI and VAT as covariates in the genetic association studies.

### Identification of 17 previously unknown loci for liver triglyceride content and 9 for liver inflammation/fibrosis by the multi-adiposity-adjusted GWAS

**Fig. 1:**
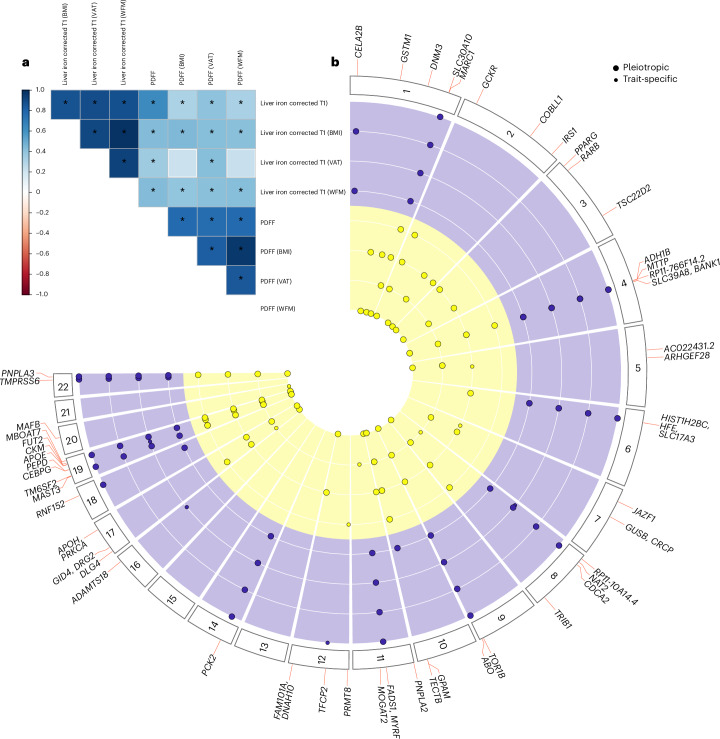
Overview of the identified loci for liver triglycerides and inflammation/fibrosis by the multi-adiposity-adjustment GWAS. **a**, Genetic correlation among different multi-adiposity-adjusted PDFF and liver iron corrected T1 was estimated using LD score regression analysis. The asterisks denote Benjamini–Hochberg false discovery rate (FDR) <0.05. The color bar represents the genetic correlation values. Detailed summary statistics for genetic correlations have been reported in Supplementary Table 3. **b**, Circular Manhattan plot of PDFF and liver iron corrected T1 for different adiposity adjustments. The association analyses were performed using REGENIE adjusting for adiposity index, age, sex, age × sex, age^2^ and age^2 ^× sex, first ten genomic principal components and array batch. Each dot represents an independent genetic locus. Yellow represents loci associated with liver PDFF and purple represents those associated with liver cT1. Large dots represent pleiotropic loci (where the association with either PDFF or liver cT1 was shared among two or more adiposity adjustments). Small dots show adiposity-trait specific associations. Loci in bold are shared among both traits irrespective of the adiposity adjustment. Only loci with a genome-wide significant *P* <5 × 10^−8^ calculated by a whole-genome regression model (Methods) are shown. *P* values were two-sided and not corrected for multiple testing among four different models (unadjusted, adjusted for BMI, WFM and VAT).

To capitalize on the independent contribution of indices of adiposity to proxies of liver triglyceride content (PDFF) and inflammation/fibrosis (cT1), we conducted eight GWAS (four GWAS per each trait), together referred to as the multi-adiposity-adjusted GWAS. Each GWAS was adjusted for a specific index of adiposity (VAT, BMI and WFM), and one unadjusted (Supplementary Table 1)^16^. Genetic heritability estimates for PDFF showed that adjustment for adiposity index explains up to 6% more heritability compared to the unadjusted model (Supplementary Table 2). For cT1, all adiposity measurements yielded similar results to the unadjusted model. These data suggest that liver triglyceride content is dependent on adiposity, whereas inflammation is less correlated. Furthermore, genetic correlations among different adiposity adjustments showed that BMI and WFM adjustments shared the largest overlap for both PDFF and cT1 (Fig. 1a and Supplementary Table 3), consistent with the epidemiological correlation.

Statistically independent genetic loci for each adiposity-adjusted GWAS were identified by linkage disequilibrium (LD) clumping^17^ and conditional analysis (Methods)^18^. Next, we performed pleiotropic analysis^19^ to identify independent genetic loci from the four adiposity adjustments. In this context, pleiotropic analysis refers to genetic loci that are shared among more than two adiposity adjustments for each liver trait (Supplementary Table 4). Specifically, we assigned the same locus number to lead variants from adiposity-adjusted GWAS (four for PDFF and four for cT1) located within 1 Mb of each other, provided that more than two GWAS lead variants were in LD (*r*^2 ^> 0.2). Finally, the strongest (lowest GWAS *P* value) association at each locus was selected as the independent lead variant for that trait (PDFF or cT1).

A total of 37 and 18 independent genetic loci for PDFF and cT1 reached the genome-wide significance level (*P* < 5 × 10^−8^ not adjusted for the number of GWAS carried out), respectively (Fig. 1b and Table 1). Multiple loci showed the strongest associations when adjusted for specific adiposity indices (Supplementary Table 5).

**Table 1:** Genome-wide significant loci for multi-adiposity-adjusted PDFF and liver iron corrected T1 in the UK Biobank

Trait	CHR	POS	Variant ID	*n*	Consequence	A1	A2	A1Freq	*β*	s.e.m.	*P*	locus	MAGMA
PDFF (WFM)	**1**	**110232983**	rs140584594	**34,367**	**missense_variant**	**A**	**G**	**0.267**	**−0.049**	**0.007**	**1.42** × **10**^−**13**^	***GSTM1***	
PDFF (BMI)	**1**	**172323134**	rs17277932	**35,146**	**intron_variant**	**A**	**G**	**0.145**	**0.049**	**0.008**	**5.91** **×** **10**^**−9**^	***DNM3***	
PDFF (VAT)	1	220970028	rs2642438	33,540	missense_variant	A	G	0.296	−0.061	0.006	7.89 × 10^−24^	*MARC1*	5.91 × 10^−14^
PDFF (BMI)	2	27730940	rs1260326	35,146	missense_variant	T	C	0.392	0.063	0.006	4.23 × 10^−25^	*GCKR*	4.51 × 10^−14^
PDFF (WFM)	2	165501927	rs5835988	34,367	intergenic_variant	TG	T	0.408	−0.046	0.006	3.25 × 10^−14^	*COBLL1*	3.58 × 10^−11^
PDFF (WFM)	2	227100579	2:227100579_TC_T	34,367	intergenic_variant	TC	T	0.348	−0.042	0.006	1.63 × 10^−11^	*IRS1*	
PDFF (WFM)	3	12393125	rs1801282	34,367	missense_variant	G	C	0.122	−0.059	0.009	6.61 × 10^−11^	*PPARG*	
PDFF (WFM)	**3**	**25484121**	rs79905393	**34,367**	**intron_variant**	**A**	**G**	**0.036**	**−0.094**	**0.016**	**2.86** × **10**^−**9**^	***RARB***	
PDFF (WFM)	**3**	**150066540**	rs62271373	**34,367**	**regulatory_region_variant**	**A**	**T**	**0.060**	**0.082**	**0.013**	**1.02** × **10**^−**10**^	***TSC22D2***	
PDFF (NA)	4	100239319	rs1229984	36,394	missense_variant	T	C	0.024	−0.165	0.023	1.26 × 10^−12^	*ADH1B*	
PDFF (VAT)	4	100503761	rs11937107	33,540	intron_variant	T	C	0.252	−0.044	0.006	4.87 × 10^−12^	*MTTP*	8.20 × 10^−12^
PDFF (BMI)	**4**	**100562374**	rs36029295	**35,146**	**intron_variant**	**GA**	**G**	**0.039**	**0.085**	**0.015**	**2.68** × **10**^−**8**^	***RP11-766F14.2***	
PDFF (VAT)	5	55808342	rs392794	33,540	intron_variant	C	T	0.254	−0.063	0.006	5.09 × 10^−23^	AC022431.2	6.83 × 10^−13^
PDFF (BMI)	**5**	**72951153**	5:72951153_GT_G	**35,146**	**intron_variant**	**G**	**GT**	**0.462**	**0.033**	**0.006**	**3.45** **×** 10^−**8**^	***ARHGEF28***	
PDFF (BMI)	6	26093141	rs1800562	35,146	missense_variant	A	G	0.077	0.062	0.011	3.27 × 10^−8^	*HFE*	
PDFF (BMI)	7	28172732	rs702814	35,146	intron_variant	C	T	0.492	0.035	0.006	3.73 × 10^−9^	*JAZF1*	
PDFF (VAT)	**7**	**65431686**	rs6955582	**33,540**	**intron_variant**	**A**	**G**	**0.451**	**−0.034**	**0.006**	**1.72** × **10**^−**9**^	***GUSB***	**1.03 ×** **10**^**−8**^
PDFF (VAT)	**8**	**25464670**	rs73221948	**33,540**	**intergenic_variant**	**T**	**G**	**0.294**	**0.046**	**0.006**	**3.05** × **10**^−**13**^	***CDCA2***	
PDFF (BMI)	8	126500031	rs28601761	35,146	intron_variant	G	C	0.420	−0.072	0.006	1.63 × 10^−32^	*TRIB1*	4.72 × 10^−7^
PDFF (VAT)	9	132566666	rs7029757	33,540	non_coding_transcript_exon_variant	A	G	0.096	−0.063	0.009	3.15 × 10^−11^	*TOR1B*	
PDFF (VAT)	10	113910721	rs1129555	33,540	3_prime_UTR_variant	A	G	0.273	0.062	0.006	2.52 × 10^−23^	*GPAM*	4.04 × 10^−14^
PDFF (VAT)	**10**	**114024573**	10:114024573_GA_G	**33,540**	**intergenic_variant**	**G**	**GA**	**0.286**	**−0.039**	**0.006**	**2.24** × **10**^−**10**^	***TECTB***	**4.90 ×** **10**^**−9**^
PDFF (WFM)	11	823586	rs140201358	**34,367**	missense_variant	G	C	0.015	0.147	0.025	2.35 × 10^−9^	*PNPLA2*	
PDFF (VAT)	**11**	**75456134**	rs531117	**33,540**	**downstream_gene_variant**	**T**	**C**	**0.157**	**−0.042**	**0.008**	**3.91** × **10**^−**8**^	***MOGAT2***	
PDFF (NA)	**12**	**3685100**	rs1985912	**36,394**	**intron_variant**	**G**	**A**	**0.326**	**−0.043**	**0.008**	**2.67** × **10**^−**8**^	***PRMT8***	
PDFF (WFM)	**12**	**124476873**	rs12833624	**34,367**	**intron_variant**	**T**	**C**	**0.337**	**−0.039**	**0.006**	**3.37** × **10**^−**10**^	***FAM101A***	**1.01 ×** **10**^**−6**^
PDFF (VAT)	**17**	**7116853**	rs446994	**33,540**	**intron_variant**	**C**	**A**	**0.422**	**0.033**	**0.006**	**3.43** × **10**^−**9**^	***DLG4***	**1.04 ×** **10**^**−8**^
PDFF (NA)	17	17974014	rs11439486	36,394	downstream_gene_variant	T	TA	0.330	0.046	0.008	7.63 × 10^−9^	*GID4*	2.19 × 10^−7^
PDFF (VAT)	**17**	**64210580**	rs1801689	**33,540**	**missense_variant**	**C**	**A**	**0.029**	**0.099**	**0.016**	**1.63** × **10**^−**9**^	***APOH***	
PDFF (NA)	19	18229208	rs56252442	36,394	intron_variant	T	G	0.253	0.048	0.008	5.11 × 10^−9^	*MAST3*	5.13 × 10^−7^
PDFF (VAT)	19	19379549	rs58542926	33,540	missense_variant	T	C	0.075	0.292	0.011	4.7 × 10^−168^	*TM6SF2*	5.00 × 10^−10^
PDFF (VAT)	**19**	**33834096**	rs73026242	**33,540**	**downstream_gene_variant**	**G**	**A**	**0.072**	**0.060**	**0.011**	**3.59** × 10^−**8**^	***CEBPG***	
PDFF (BMI)	19	45411941	rs429358	35,146	missense_variant	C	T	0.151	−0.102	0.008	2.92 × 10^−35^	*APOE*	1.67 × 10^−15^
PDFF (NA)	**19**	**45830763**	rs344790	**36,394**	**upstream_gene_variant**	**A**	**C**	**0.421**	**0.043**	**0.007**	**2.68** × 10^−**9**^	***CKM***	
PDFF (VAT)	19	54671421	rs60204587	33,540	intron_variant	A	G	0.426	0.045	0.006	1.42 × 10^−15^	*MBOAT7*	5.27 × 10^−15^
PDFF (WFM)	**20**	**39142516**	rs2207132	**34,367**	**intergenic_variant**	**A**	**G**	**0.034**	**0.092**	**0.016**	**1.73** × 10^−**8**^	***MAFB***	
PDFF (VAT)	22	44324730	rs738408	33,540	synonymous_variant	T	C	0.214	0.237	0.007	1.7 × 10^−269^	*PNPLA3*	1.38 × 10^−14^
cT1 (BMI)	**1**	**15810346**	rs12130283	**29,312**	**intron_variant**	**C**	**T**	**0.484**	**0.045**	**0.007**	**5.33** × 10^−**10**^	***CELA2B***	**6.91 ×** **10**^**−8**^
cT1 (BMI)	1	220100497	rs759359281	29,312	splice_polypyrimidine_tract_variant	C	CA	0.055	0.129	0.016	4.74 × 10^−15^	*SLC30A10*	
cT1 (VAT)	4	103188709	rs13107325	27,782	missense_variant	T	C	0.070	0.556	0.015	<4.94 × 10^−324^	*SLC39A8*	2.01 × 10^−18^
cT1 (VAT)	6	25878848	rs55925606	27,782	intron_variant	G	A	0.073	−0.149	0.015	1.07 × 10^−24^	*SLC17A3*	3.32 × 10^−9^
cT1 (VAT)	**8**	**9173209**	rs7012637	**27,782**	**intron_variant**	**A**	**G**	**0.472**	**0.043**	**0.007**	**9.39** × **10**^−**9**^	***RP11-10A14.4***	
cT1 (BMI)	**8**	**18272881**	rs1495741	**29,312**	**regulatory_region_variant**	**G**	**A**	**0.219**	**−0.064**	**0.009**	**4.5** × **10**^−**13**^	***NAT2***	
cT1 (NA)	**9**	**136149830**	rs532436	**30,481**	**intron_variant**	**A**	**G**	**0.183**	**0.079**	**0.010**	**7.47** × **10**^−**15**^	***ABO***	
cT1 (WFM)	10	113921825	rs2792735	28,638	intron_variant	G	A	0.277	0.046	0.008	4.05 × 10^−8^	*GPAM*	4.81 × 10^−7^
cT1 VAT)	**11**	**61549025**	rs174533	**27,782**	**intron_variant**	**A**	**G**	**0.351**	**0.052**	**0.008**	**2.49 ×** **10**^**−11**^	***MYRF***	**3.51 ×** **10**^**−7**^
cT1 (NA)	**12**	**51511269**	rs59372312	**30,481**	**intron_variant**	**G**	**A**	**0.067**	**−0.088**	**0.016**	**2.33 ×** **10**^**−8**^	***TFCP2***	
cT1 (BMI)	14	24572932	rs111723834	29,312	missense_variant	A	G	0.016	0.383	0.029	1.7 × 10^−39^	*PCK2*	9.99 × 10^−8^
cT1 (VAT)	**16**	**77423427**	rs2454933	**27,782**	**intron_variant**	**T**	**C**	**0.082**	**0.075**	**0.014**	**3.69 ×** **10**^**−8**^	***ADAMTS18***	
cT1 (NA)	**18**	**59323966**	18:59323966_CTT_C	**30,481**	**intron_variant**	**C**	**CTT**	**0.287**	**−0.050**	**0.009**	**1.28 ×** **10**^**−8**^	***CDH20***	
cT1 (WFM)	19	19379549	rs58542926	28,638	missense_variant	T	C	0.074	0.141	0.014	4.22 × 10^−23^	*TM6SF2*	4.46 × 10^−7^
cT1 (VAT)	**19**	**33890838**	rs10406327	**27,782**	**intron_variant**	**G**	**C**	**0.477**	**−0.042**	**0.007**	**1.24 ×** **10**^**−8**^	***PEPD***	**1.84 ×** **10**^**−06**^
cT1 (BMI)	19	49206172	rs516246	29,312	intron_variant	C	T	0.489	−0.053	0.007	3.67 × 10^−13^	*FUT2*	1.00 × 10^−13^
cT1 (VAT)	22	37469192	rs6000553	27,782	intron_variant	A	G	0.466	0.070	0.007	7.53 × 10^−21^	*TMPRSS6*	4.81 × 10^−12^
cT1 (BMI)	22	44324727	rs738409	29,312	missense_variant	G	C	0.214	0.120	0.009	3.2 × 10^−41^	*PNPLA3*	2.85 × 10^−13^

The association between common genetic variants and PDFF under different adiposity adjustments was performed using REGENIE adjusting for adiposity index, age, sex, age × sex, age^2^ and age^2 ^× sex, first ten genomic principal components and array batch. Each adiposity adjustment is shown in parentheses. Genomic loci in bold represent the previously unknown loci identified in the present work. The locus column shows the nearest gene to the lead variant (from COJO analysis) at each locus. MAGMA column shows significant gene-based associations at each locus exceeding Bonferroni threshold (*P* < 2.65 × 10^−6^). Sample size used in each GWAS has been shown in column N. *P* values were not corrected for multiple testing among four different models (unadjusted, adjusted for BMI, WFM and VAT). NA, unadjusted; MAGMA, multi-marker analysis of genomic annotation.

We found 17 and 9 previously unknown genetic loci associating with PDFF and cT1, respectively (Methods, Table 1 and Supplementary Table 6). Four loci (*PNPLA3*, *TM6SF2*, *GPAM* and *HFE/SLC17A3*) were associated with both traits with at least one adiposity adjustment; however, only *PNPLA3* and *TM6SF2* loci were associated with both traits at a genome-wide level irrespective of the adjustment (Fig. 1b).

### Identification of the putative causal loci associated with liver traits

To identify the putative causal loci, we fine-mapped the independent genome-wide significant loci associated with adiposity-adjusted PDFF and cT1. Independent lead variants at multiple loci had a posterior inclusion probability (PIP) > 0.95, suggesting that these GWAS lead variants are causal variants (Supplementary Table 7). Notably, a missense variant on *ADH1B* (rs1229984) had a PIP of 1 at *ADH1B*, *MTTP* and *RP11-766F14.2* loci, suggesting that the observed effect from all three loci may derive from the same putative causal variant. In fact, *ADH1B* rs1229984 and *MTTP* rs11937107 have a D′ of 1 in Europeans^20^ (Supplementary Table 7).

To examine whether the set of independent variants could potentially perturb the gene expression patterns of nearby genes, we performed a Bayesian colocalization (Methods). We were able to colocalize 13 and 7 GWAS signals with at least one eQTL evidence for PDFF and cT1, respectively (Supplementary Table 8).

### Functional analyses of independent loci associated with liver traits

Independent genetic loci for adiposity-adjusted PDFF and cT1 were mapped to genes and ranked using multiple approaches (Methods). Out of 37 and 18 independent loci for PDFF and cT1, respectively, the majority (31 and 12) loci had the highest rank for the nearest genes (Supplementary Table 9). For the remaining loci, multiple candidate genes were found. To gain a deeper understanding of the biological implications of genome-wide significant loci, we conducted a functional gene-set enrichment analysis using mapped genes with the highest evidence (Supplementary Table 10). Mapped genes for PDFF were enriched in genes mostly expressed in liver and they were involved in lipid metabolism (Supplementary Table 10a and Supplementary Fig. 1a). Conversely, mapped genes for liver iron corrected T1 were enriched in metal ion metabolism (Supplementary Table 10b and Supplementary Fig. 1b).

### Previously unknown genetic loci, liver and metabolic traits

**Extended Data Fig. 2:**
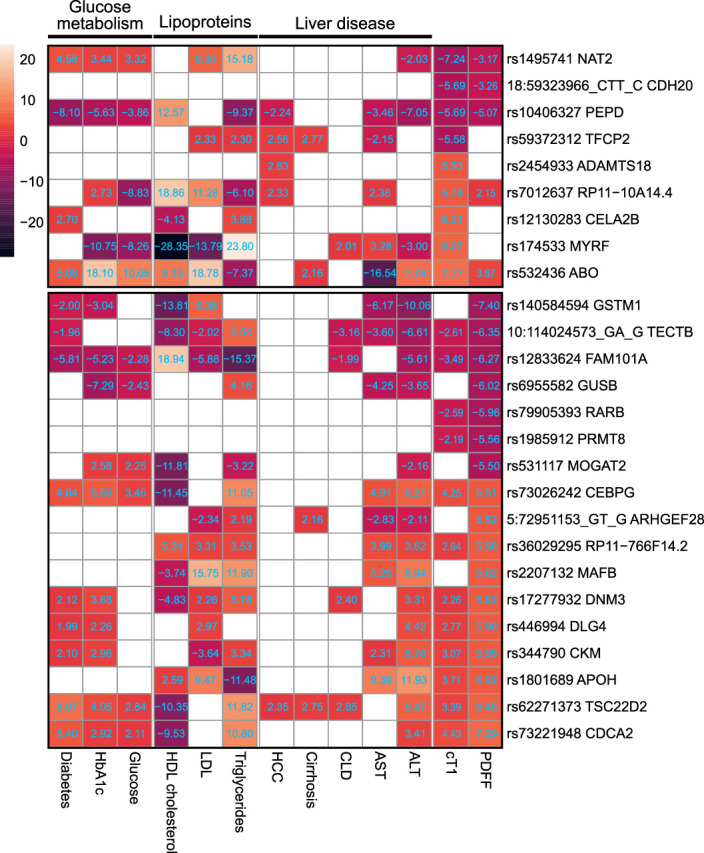
Previously unknown genetic loci were associated with liver disease and metabolic traits. Heatmap of the Z-score of associations for the effect (risk) allele between previously unknown genetic *loci* and liver or metabolic-related traits (columns) in n=397,780 UKBB participants after excluding individuals with available PDFF or liver iron corrected T1 (n=36,748). The association analyses were performed by linear or logistic regression analysis using REGENIE and adjusted for adiposity index, age, sex, age×sex, age^2^ and age^2^×sex, first 10 genomic principal components and array batch. Upper and lower boxes correspond to liver iron corrected T1 and PDFF genetic *loci*, respectively. Full summary statistics have been reported in Supplementary Table 11. P values were two-sided and not corrected for multiple hypothesis testing. VAT: Visceral adipose tissue; WFM: Whole-body fat mass (kg/m2); cT1: liver iron corrected T1; PDFF: proton density fat fraction; CLD: chronic liver disease.

Given the causal relationship between liver triglyceride content and inflammation/fibrosis, we examined the association of the previously unknown variants identified by PDFF with cT1 and vice versa. Notably, most of the variants were associated with both traits and directionally concordant (Extended Data Fig. 2). This is consistent with the notion that liver triglyceride content causes inflammation^21^. A total of 5 (29 %) and 4 (44 %) loci were associated with either PDFF or cT1, suggesting a specificity of the effect on lipid or inflammation pathways. Furthermore, we examined the association between these previously unknown variants and indices of liver damage, fibrosis and liver disease (Extended Data Fig. 2 and Supplementary Table 11). More than 80% of variants associated with PDFF also associated with alanine aminotransferase (ALT) (one-sided Fisher’s exact test *P* = 0.028); however, there was no significant correlation between PDFF and cT1 loci associations with aspartate aminotransferase (one-sided Fisher’s exact test *P* = 0.613). Most variants were associated with plasma lipoproteins and glucose metabolism traits, including diabetes (Extended Data Fig. 2 and Supplementary Table 11).

### Indices of adiposity contribute differentially to the association between genetic variants and liver triglycerides

Adiposity is a well-known risk factor for MASLD and there is no evidence on causal impact of SLD on adiposity^22^. Hence, it is safe to assume that adjusting for adiposity does not suffer from collider bias. Therefore, we hypothesized that the association between PDFF or cT1 and genetic loci depends on the measures of adiposity. To explore this, we performed different statistical analyses (Methods). As reported in Supplementary Tables 12 and 13, while the overall associations are consistent across multi-adiposity-adjusted GWAS, some loci display different associations depending on the adiposity adjustment.

For instance, we found no association between PDFF and rs73026242*CEBPG* with BMI or WFM adjustments, but a strong genome-wide association with VAT adjustment. This locus has a strong association with VAT but in the opposite direction to that of PDFF, and mediation analysis suggests an inconsistent mediation, namely, a partial mediation in the opposite direction. This locus has been recently linked with visceral to abdominal subcutaneous fat ratio^15^. Our gene mapping suggests that *CEBPA*, known for its role in adipogenesis through PPARγ, is the potential causal gene (Supplementary Table 9a)^23^.

Conversely, while the *PPARG* locus shows evidence of interaction with all three adiposity measures, a putative inconsistent effect was only observed for WFM adjustment. This variant decreases PPARγ activity^24^ and confers a protection against diabetes^25,26^. While the contribution of this variant to SLD is a matter of debate^27^, we observed a modest positive association with WFM. Another interesting finding is *FAM101A* locus, where there is a nominally significant association with BMI/WFM and VAT but in opposite directions. Hence, adjusting for VAT mitigated the association (*P*^VAT^ = 0.01). The top-ranked gene at this locus, *CCDC92*, has been shown to play a role in insulin resistance and subcutaneous adipose and peripheral fat^28^. On the other hand, we also encounter the opposite scenario, where adiposity may act as a positive partial mediator, as for the *PRMT8*, *MAST3* and *CKM* loci.

For cT1, while most loci have consistent associations over different adiposity adjustments, the mediation analysis suggests a putative inconsistent effect for VAT-adjusted model at *PEPD* locus. The top-ranked gene at this locus is *CEBPA*; however, whether the putative mechanism is similar to the one described above for liver triglyceride content is unclear because *PEPD* has a similar rank and is associated with diabetes and adiposity^29^.

Finally, we performed sensitivity analysis to examine the robustness of mediation analysis to unmeasured confounders, and observed a strong (for PDFF, *ρ* = 0.6) and moderate (for cT1, *ρ* = 0.4) robustness of mediation estimates to the sequential ignorability assumption^30^. Given the causality assumptions in mediation analysis, these potential mechanisms should be considered with caution. Furthermore, due to the statistical equivalence of mediation and confounding effects^31^, the observed inconsistent mediations may be interpreted as negative confounding effects that may enable the discovery of unknown genetic loci.

Considering this extensive body of evidence, measures of adiposity contribute to the association between genetic loci and proxies of liver triglyceride content (PDFF) and inflammation/fibrosis (cT1), thus supporting our multi-adiposity-adjusted GWAS approach.

### The association between six previously unknown loci and liver triglyceride content was replicated in independent cohorts

**Fig. 2:**
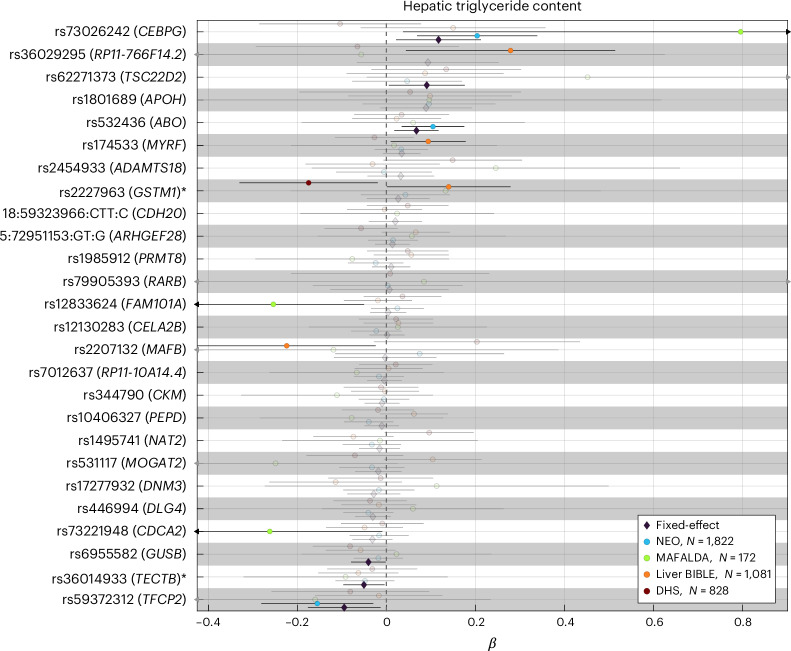
The association between six previously unknown loci and hepatic triglyceride content in independent cohorts. The association between each genetic variant and rank-based inverse normal transformed hepatic triglyceride content was performed using a linear regression analysis adjusted for age, sex, age^2^, age × sex, age^2^ × sex (shown as circles). Proxy variants were used for variants not available in the replication cohorts (*r*^2^ > 0.4 within a window of 1.5 Mb around each lead variant in the UK Biobank) as marked with an asterisk. Pooled effect estimates were calculated using inverse-variance-weighted fixed-effect meta-analysis (shown as diamonds). Genomic loci in bold are those with a *P* value <0.05 in the fixed-effects model. Error bars represent the 95% confidence intervals from the regression models or meta-analysis. Full summary statistics have been reported in Supplementary Table 14. *P* values are two-sided and not adjusted for multiple testing. NEO, Netherlands Epidemiology of Obesity study; DHS, Dallas Heart Study.

Based on the strong genetic correlation between PDFF and cT1, to validate the previously unknown SNPs, we meta-analyzed the association between all the previously unknown 26 variants and liver triglyceride content in 3,903 individuals of European ancestry from four independent cohorts (Fig. 2 and Supplementary Table 14). We were able to replicate the association between six of the previously unknown loci (*CEBPG*, *TSC22D2*, *ABO*, *GUSB*, *TECTB* and *TFCP2*) and liver triglyceride content. The direction of the association in the replication cohort was consistent with the discovery cohort.

### Partitioned polygenic risk scores identify a steatotic liver-specific disease and a systemic MASLD

Triglyceride secretion is a key mechanism regulating hepatocyte triglyceride homeostasis. Triglyceride secretion is mediated by very-low-density lipoprotein (VLDL) secretion that in fasting conditions are proxied by circulating triglyceride levels. Variants in genes hampering VLDL secretion, including *APOB*, *MTTP*, *TM6SF2* and *PNPLA3*, cause retention of liver triglycerides mirrored by lower circulating lipoproteins. Carriers of these variants have an increased risk of MASLD and lower risk for cardiovascular disease due to lower lipoproteins^32–34^.

**Extended Data Fig. 3:**
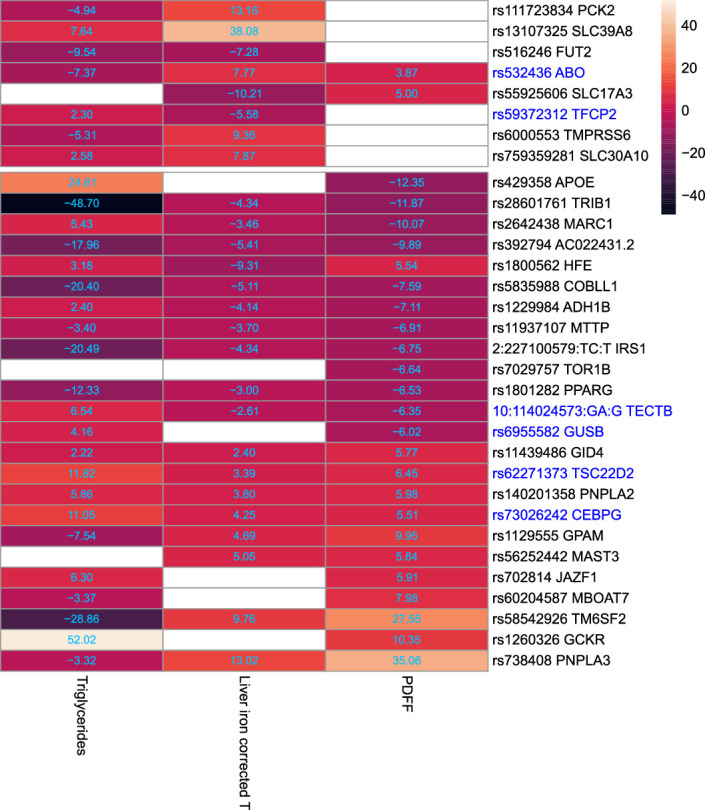
Association between 26 previously known and 6 previously unknown replicated genetic *loci* and circulating triglycerides in the UK Biobank. The heatmap shows the Z-score of associations for the effect (risk) allele in Europeans (n=397,780) after excluding individuals with available PDFF or liver iron corrected T1 (n=36,748). The association was performed by linear regression analysis using REGENIE and adjusted for adiposity index, age, sex, age×sex, age^2^ and age^2^×sex, first 10 genomic principal components and array batch. Upper and lower boxes correspond to liver iron corrected T1 and PDFF genetic *loci*, respectively. Previously unknown replicated genetic *loci* have been marked in blue. Full summary statistics have been reported in Supplementary Table 15. P values were two-sided and not corrected for multiple hypothesis testing.

Based on this mechanism, we allocated the previously unidentified replicated and the previously known variants into two pPRSs: (1) a group showing discordant association between PDFF and circulating triglycerides (*n* = 10), suggesting that liver triglyceride content is primarily influenced by liver retention, and (2) a group with concordant associations (*n* = 13), indicating that liver triglyceride content may result from an increase in uptake, synthesis of energy substrates or a reduction in β-oxidation (Methods, Extended Data Fig. 3 and Supplementary Table 15). The variance explained by the discordant pPRS was higher than the concordant pPRS mirroring the fact that discordant pPRS is composed of *PNPLA3* and *TM6SF2* variants (Supplementary Table 16).

**Fig. 3:**
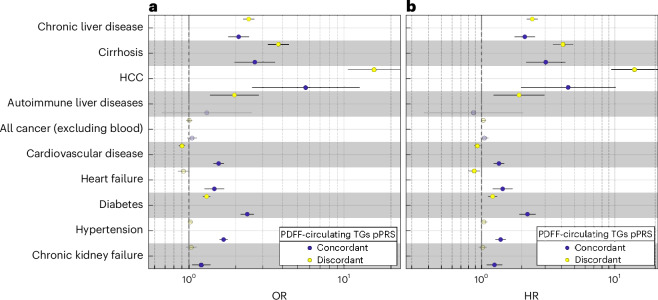
Partitioned polygenic risk scores identify a steatotic liver-specific disease and a systemic MASLD. **a**,**b**, The case–control (**a**) and prospective (**b**) association between two PDFF-circulating TGs pPRS and liver-related, cardiometabolic and chronic kidney failure traits in the UK Biobank. Effect plot of the association between concordant and discordant PDFF-circulating TGs PRS with each disease was tested using either logistic (**a**) or Cox proportional hazard (**b**) regression analysis adjusted for BMI, age, sex, age × sex, age^2^ and age^2^ × sex, first ten genomic principal components and array batch. The *x* axis shows either the odds ratio (OR) or hazard ratio. All association analyses have been performed after excluding individuals with available PDFF (*n* = 36,394). Error bars represent the 95% confidence intervals from the regression models. Full summary statistics have been reported in Supplementary Table 18. *P* values were two-sided and not corrected for multiple hypothesis testing. TG, triglyceride.

Both pPRSs were associated with an increased risk of MASLD with the largest association being with hepatocellular carcinoma (HCC); however, the association was stronger for the discordant pPRS (Fig. 3a, Supplementary Fig. 2 and Supplementary Tables 17 and 18). Of note, only the discordant pPRS was associated with autoimmune liver diseases.

Discordant pPRS was associated with a decreased risk of cardiovascular, whereas concordant PRS was associated with a substantial increased risk of cardiovascular disease and heart failure. Both pPRSs conferred an increased predisposition to diabetes, suggesting that hepatic triglyceride accumulation predisposes to diabetes irrespective of the underlying cause. Conversely, the larger effect size of the concordant pPRS for diabetes despite the lower effect on liver triglyceride content would suggest that the association of diabetes in the concordant pPRS is not mediated by liver damage. In the case of hypertension and chronic kidney failure, discordant pPRS showed no association, whereas the concordant pPRS increased the risk of both diseases; however, when we adjusted for hypertension, the association with chronic kidney failure was no longer significant, whereas the other associations remained (Supplementary Table 18). Further adjustment for diabetes, total cholesterol and alcohol intake did not change the results (Supplementary Table 18). The prospective risk conferred by the pPRS to develop liver and cardiometabolic disease in the UK Biobank was virtually identical (Fig. 3b and Supplementary Table 18).

Functional gene-set enrichment analysis for both pPRSs also revealed a distinct metabolic pattern. While gene sets of discordant pPRS were mostly enriched in lipid and triglyceride homeostasis (Supplementary Table 10c and Supplementary Fig. 3), concordant pPRS gene sets were enriched in insulin receptor signaling and glucose homeostasis pathways, overall consistent with an impact on stimulation of de novo lipogenesis (Supplementary Table 10d and Supplementary Fig. 3).

In addition to our hypothesis-driven approach, we performed an unsupervised soft clustering approach^35^ (Methods). Of 1,000 iterations, 90% converged to two clusters and 10% to one cluster. One genetic locus, rs738408*PNPLA3*, appeared in both clusters^36^ (Supplementary Fig. 4). We used two top-weighted traits to name the clusters: (1) low-density lipoprotein (negative)/triglycerides (negative); and (2) triglycerides (positive)/ALT (positive). When examining the association of pPRS clusters with the same outcomes, we observed a similar dissociation to the PDFF-circulating TGs pPRS; however, differences in the risk of diseases defining the two types of MASLD were larger in our hypothesis-driven approach (Supplementary Table 19). This may be attributed to the soft clustering feature of the Bayesian non-negative matrix factorization (bNMF) algorithm, where rs738408*PNPLA3* was included in both clusters (Supplementary Fig. 5).

When comparing plasma biomarkers between individuals in the upper and lower quartiles of pPRS, those in the upper quartile of discordant pPRS had the largest differences in lipoprotein levels (Supplementary Table 20), consistent with the protective effect of the liver-specific subtype compared to the systemic subtype. In addition, individuals in the top quartile of concordant pPRS had higher lipoproteins and blood pressure and lower creatinine levels, consistent with the increased risk of cardiovascular disease, heart failure and kidney failure.

### Sex-specific effect of the association between pPRS and the feature of cardiometabolic syndrome

Liver diseases have a different prevalence between males and females. For instance, HCC is more frequent in men while liver autoimmune disease is more prevalent in females. Moreover, there are sex-specific differences in carriers of the *PNPLA3* rs738409 between males and females^37^. Therefore, we examined the association between the two pPRS and cardiometabolic syndrome stratified by sex. Results of the stratified analyses are consistent with the pooled analyses, with the following exceptions: (1) HCC is associated with the concordant pPRS only in males; (2) heart failure is associated with protection in the discordant pPRS specifically in females; and (3) chronic kidney failure is increased in the concordant pPRS only in males (Supplementary Fig. 6 and Supplementary Table 21).

### mRNA expression of loci from the liver-specific PRS is more abundant in the liver

**Fig. 4:**
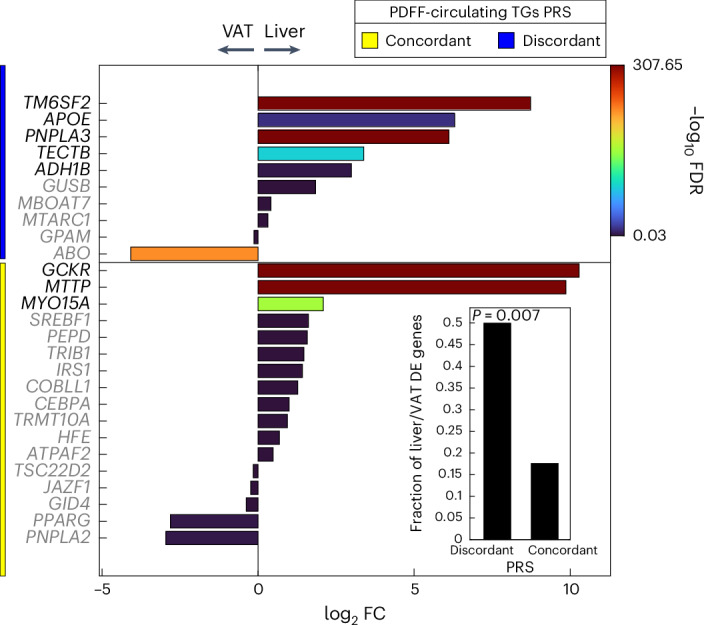
mRNA expression of loci from the liver-specific (discordant) polygenic risk score is more abundant in the liver compared to the visceral adipose tissue. Differential expression analysis of paired liver and VAT bulk RNA-seq data for mapped gene sets of concordant and discordant pPRS. The lower right bar plot shows the fraction of upregulated differentially expressed (DE) genes in the liver compared to VAT. The enrichment of pPRS with upregulated DE genes in the liver was calculated using one-sided Fisher’s exact test. FC, fold change.

We further examined the messenger RNA expression of mapped genes within concordant and discordant pPRS using paired bulk RNA-seq of liver (*n* = 244) and VAT (*n* = 261) from participants with obesity from the MAFALDA cohort (Molecular Architecture of Fatty Liver Disease in Patients with Obesity Undergoing Bariatric Surgery study). Notably, only the mapped genes of discordant pPRS showed a significant overlap with upregulated differentially expressed genes in the liver (one-sided Fisher’s exact test, *P* = 0.007; Fig. 4). Given the tight interplay between VAT and liver in the MASLD, this finding suggests a liver-specific nature of discordant pPRS compared to its metabolic counterpart, concordant pPRS.

## Discussion

The main findings of this study are (1) the identification of previously unknown loci associated with SLD; and (2) the identification of two distinct types of MASLD, namely, a liver-specific and a systemic type.

BMI, as a proxy of adiposity, amplifies the genetic predisposition to SLD given by common variants^14,22^; however, BMI does not consider body fat distribution and body composition. To identify previously unknown genetic loci associated with SLD, we compared a range of measurements of adiposity finding that VAT volume, WFM and BMI were the best independent predictors.

Next, we performed multi-adiposity-adjusted GWAS on PDFF and iron corrected T1, as a measure of liver triglyceride content and inflammation/fibrosis. Our data demonstrate that adiposity indices may confound association between genetic variants and liver triglyceride content and inflammation/fibrosis. By using this approach, we identified 17 previously unknown genetic loci for liver triglyceride content and 9 for liver inflammation, and replicated 6 of these loci in four independent cohorts.

The heritability of liver triglyceride content was influenced by the multi-adiposity adjustment explaining in the best-case scenario approximately 6% more heritability compared to the unadjusted; however, for inflammation this was not the case, suggesting that heritability of inflammation is not directly influenced by adiposity. We have previously demonstrated a causal link between liver triglyceride content and increased risk of liver inflammation using Mendelian randomization^21^. This finding is further supported by our observation that approximately 80% of the genetic loci associated with PDFF also associate with cT1 in the same direction.

Intrahepatocyte triglyceride homeostasis is governed by three fundamental mechanisms: triglyceride synthesis, lipoprotein secretion and energy substrate utilization. Hindering lipoprotein secretion causes liver triglyceride accumulation by retention. Indeed, loss-of-function variants in *TM6SF2* and *PNPLA3* cause liver triglyceride retention by reducing lipoprotein secretion^38,39^. Consistently with hepatic lipoprotein retention, carriers of these variants have lower risk for cardiovascular disease due to the lower circulating lipoproteins^32,40^.

Therefore, we generated two pPRSs: one composed of variants in which the association between liver triglyceride content and circulating triglycerides were discordant and one in which they were concordant. ‘Partitioned’ polygenic scores may elucidate disease pathogenesis and capture specific signatures driving the individual disease progression, hence providing a framework for tailored therapeutic interventions^41^.

Of note, the concordant pPRS predicts the entire spectrum of cardiometabolic disease. On the contrary, the discordant pPRS is associated with liver disease mirrored by protection from cardiovascular disease due to lipoprotein retention, despite a marginal increase in the risk of diabetes. The liver specificity of the discordant pPRS is further supported by a higher mRNA expression of the genes composing this score in liver versus visceral adipose paired biopsies from individuals with obesity. We additionally generated pPRSs using hypothesis-free soft clustering^35^, which was very similar to our hypothesis-driven approach.

**Extended Data Fig. 4:**
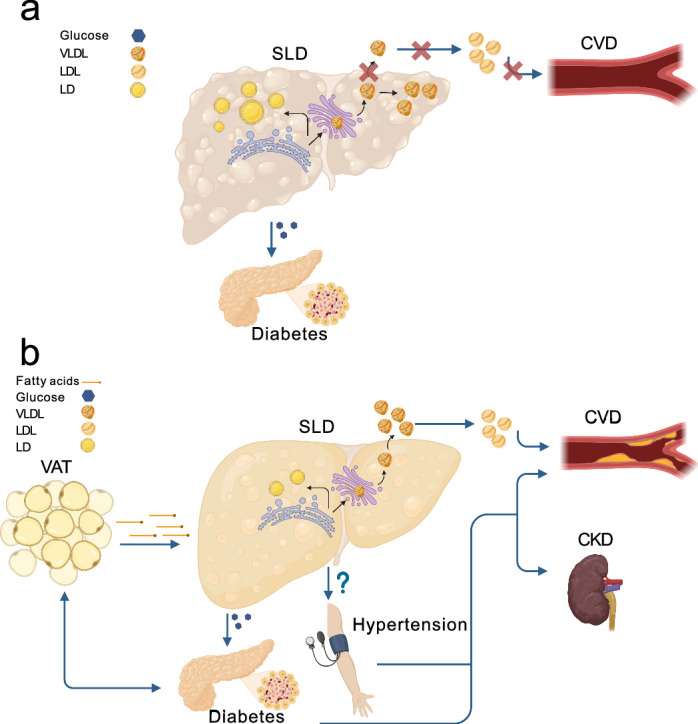
Putative model of the two different types of MASLD. **a**) In the steatotic liver-specific disease, the primary increase in the liver triglyceride content is due to the hepatic retention of very low-density lipoproteins (VLDL). This retention is causally related to liver inflammation, fibrosis, and hepatocellular carcinoma. In this type of MASLD, the higher risk of diabetes is due to the degree of liver fibrosis, while the lower risk of cardiovascular disease (CVD) to lipoprotein retention. **b**) In the systemic MASLD, the liver is entwined in the crosstalk among metabolic organs. In this type of MASLD, a dysfunctional visceral adipose tissue may increase the diabetes risk and may release free fatty acids that are incorporated into triglycerides in the hepatocytes causing liver steatosis. In turn, liver steatosis causes an overproduction of VLDL with a subsequent increase in circulating low-density lipoproteins (LDL) resulting in a higher risk of CVD. Additionally, the systemic MASLD associates with an increased blood pressure resulting in kidney failure and further increasing the CVD risk. This figure was created with BioRender.com. CKD: chronic kidney disease (failure); VAT: visceral adipose tissue; LD: lipid droplets.

Our data suggest the presence of at least two distinct types of MASLD with specific disease-causing molecular mechanisms: one specific for the liver and one systemic and entwined with cardiometabolic syndrome (Extended Data Fig. 4). Understanding the molecular mechanisms underlying these components may allow us to find effective treatments for MASLD and cardiometabolic syndrome. Clinically, these entities reflect the presence of individuals rapidly progressing to later stages of MASLD and those with a slow-progressing MASLD associated with the entire metabolic cardiometabolic syndrome. These types of MASLD may account for the disease heterogeneity and help explain why several drugs have failed in clinical trials to treat MASLD.

Currently Mendelian randomization studies are carried out by selecting variants associated with a trait and using them to explain the causal relationship with a different trait. In this study, the pPRS had opposite effects on cardiovascular risk, indicating that if we had pooled the variants together, we may have nullified the association. Therefore, our findings support the notion that the pPRSs constructed by integrating variants into physiological pathways may allow clarifying the heterogeneity of disease pathogenesis. Ultimately, this will lead to precision medicine, improving outcome prediction and therapy.

A strength of this study is that the partitioning of the PRS was based on a hypothesis-driven approach with solid knowledge of intracellular lipid homeostasis. While the finding on cardiovascular disease may be expected, the associations with hypertension and diabetes were not granted. Alcohol consumption may have an additive effect on SLD and cardiovascular disease. Alcohol is converted into triglycerides in hepatocytes and alcoholic and nonalcoholic SLD share common genetic determinants, suggesting common disease-causing mechanisms^42^. Therefore, we did not exclude individuals based on alcohol consumption. Nonetheless, sensitivity analyses showed that adjusting for alcohol did not change the results. Finally, we obtained similar results by using a completely different approach, namely, unsupervised clustering, in cohorts of individuals in whom liver biopsy was available^43^.

A limitation of our study is that the identified and replicated genetic loci were based on study cohorts of European ancestry limiting the applicability in non-Europeans. Future studies are warranted to validate these loci and the pPRS in non-European populations. Furthermore, while we performed genetic colocalization and enrichment analyses, the functional implications of the identified loci are yet to be established by in vitro and in vivo experiments.

In conclusion, we identified six previously unknown loci associated with SLD and two distinct types of SLD, namely, one that is liver specific and another that is entwined with the full spectrum of cardiometabolic syndrome.

## Methods

### UK Biobank

The UK Biobank study has recruited over 500,000 participants aged between 40 and 69 years across the United Kingdom between 2006 and 2010, with extensive phenotypic and genetic data^44^. The UK Biobank received ethical approval from the National Research Ethics Service Committee North West Multi-Centre Haydock (reference 16/NW/0274). Data used in this study were obtained under application number 37142. European ancestry was defined previously^12^ by removing outliers using genomic principal components. Additionally, participants were excluded if they fell into any of these categories: (1) more than ten putative third-degree relatives; (2) a mismatch between self-reported and genetically inferred sex; (3) putative sex chromosome aneuploidy; (4) heterozygosity and missingness outliers; and (5) withdrawn consent^44^.

### Genotypes and imputation

UK Biobank participants were genotyped using two highly similar (>95% overlap) genotyping arrays, which were then imputed centrally by the UK Biobank based on the 1000 Genomes Project phase 3, UK 10K haplotype and Haplotype Reference Consortium reference panels. Starting from approximately 97 million variants, we kept only 9,356,431 variants with a minor allele frequency (MAF) > 1%, imputation quality (INFO) score > 0.8 and Hardy–Weinberg equilibrium *P* > 1 × 10^−10^ (refs. ^12,44^).

### Definition of traits

We used adiposity measures directly provided by the UK Biobank, including VAT (data field 22407), WFM (data field 23100) and impedance of whole body (data field 23106). WHR was calculated by dividing the waist-to-hip circumference. MRI-derived PDFF and liver iron corrected T1 (cT1) were provided directly by the UK Biobank (data fields 40061 and 40062). The details of liver MRI protocols can be found elsewhere^45^. In brief, individuals were scanned using a Siemens 1.5T Magnetom Aera. Two sequences were then used for data acquisition, a multiecho-spoiled gradient-echo and a modified look locker inversion sequence (ShMOLLI) for PDFF and cT1, respectively^45^. The definition of binary traits can be found in Supplementary Table 22.

### Phenotypic prediction models

To address the multicollinearity between different measures of adiposity and to verify their contribution in predicting PDFF and cT1 values, we fit penalized linear regression models and carried out a model selection in a tenfold nested cross-validation (CV) using Least Absolute Shrinkage and Selection Operator (LASSO), Ridge and Elastic Net. LASSO penalizes the regression model using the L1-norm, effectively reducing the influence of non-contributing features to zero. On the other hand, Ridge regression utilizes the L2-norm, allowing it to shrink regression coefficients toward zero. Elastic Net combines elements of both LASSO and Ridge by incorporating both L1 and L2 penalties through a mixing parameter *α*.

To conduct the CV process, the dataset was initially divided into training (80%) and test (20%) sets. Within the training set, the outer CV assessed the performance of each model, while the inner CV was utilized for hyperparameter tuning. This tuning was accomplished by minimizing the mean squared error across a grid of *α* and shrinkage values in each fold of the outer CV. The best performing model with the lowest mean squared error was then trained on the entire training set within a tenfold CV framework. Subsequently, its performance was evaluated using the remaining test set. Finally, the model with the optimal set of hyperparameters, determined in the previous step, was fitted to the entire dataset for final evaluation^46^. Adiposity indices were standardized before the training, whereas PDFF and cT1 values were rank-based inverse normal transformed. All models were adjusted for age, sex, age^2^, age × sex and age^2^ × sex. All analyses were performed in MATLAB (MathWorks) R2023a.

### Genome-wide association analysis

The association between 9 million imputed common variants and PDFF or cT1 under different adiposity adjustments under an additive genetic model was performed using a whole-genome regression model as implemented in REGENIE (v.3.2.8)^16^. The analysis was adjusted for age at MRI, sex, age^2^, age × sex, age^2^ × sex, the first ten principal components (PCs) of ancestry, genotyping array and adiposity index, where adiposity index was VAT, WFM, BMI or no adiposity adjustments.

Similarly, we tested the association between independent lead variants from multi-adiposity-adjusted GWAS of PDFF and cT1, and other binary or continuous metabolic traits using either a logistic or a linear whole-genome regression model in REGENIE and adjusted for the same set of covariates, including consistent adiposity adjustments. Individuals with an available PDFF or cT1 measurements were excluded before the association analysis (*n* = 36,748). In cases where the trait was measured at baseline, we used WHR instead of VAT adjustment, as the latter was not available at baseline. To fit the whole-genome regression model in step 1 of REGENIE, a subset of directly genotyped common variants (MAF > 1%) was used. After excluding variants on long-range LD and major histocompatibility complex (MHC) regions, variants with a missingness <0.01 and with Hardy–Weinberg equilibrium *P* > 1 × 10^−15^ were retained. Finally, 146,833 markers left following an LD pruning with a window of 500,000 base pairs and pairwise *r*^2 ^< 0.1 (ref. ^17^). Continuous traits were rank-based inverse normal transformed before the analyses.

### Identifying independent variants

We first performed LD clumping (PLINK v.1.90b6.26 parameters: –clump-p1 5 × 10^−8^ –clump-r2 0.01 –clump-kb 1,000, after excluding individuals with third-degree or closer relatives^17,44^) to identify approximately independent loci. Next, to detect statistically independent variants, we conducted approximate step-wise model selection in conditional and joint multiple-single nucleotide polymorphism (SNP) analysis implemented in Genome-wide Complex Trait Analysis (GCTA-COJO^18^, v.1.94.0), with an LD window of 10 Mb and using 50,000 randomly selected unrelated Europeans from the UK Biobank for in-sample LD structure, as described previously^12^. To examine whether the identified genetic loci were previously reported, we searched the NHGRI-EBI GWAS catalog database^47^ in a window of 1 Mb around each lead variant.

### Estimating heritability and genetic correlations

SNP heritability and confounding bias were estimated with LD score regression analysis (LDSC; v.1.0.1, https://github.com/bulik/ldsc/)^48^ using the baseline LD model (v.2.2; https://data.broadinstitute.org/alkesgroup/LDSCORE/), containing 97 annotations, including functional annotations and MAF-/LD-dependent architectures^49^. Similarly, pairwise genetic correlations were calculated using LDSC analysis^48^ after excluding variants in the MHC region (chromosome 6, 25–34 Mb) due to the complex LD structure. Trait pairs with a Benjamini−Hochberg FDR < 0.05 were considered to have a significant genetic correlation. In all analyses, we set the LDSC parameter chisq-max to an arbitrary large number (99,999) to keep large-effect associations.

### Pleiotropy analysis

We evaluated whether the independent genome-wide significant loci, adjusted for different adiposity measures, were specific to each adiposity measure, common between PDFF and cT1 GWAS or shared across both. Therefore, if two independent lead variants within 1 Mb of each other were in LD (*r*^2 ^> 0.2), they were assigned the same locus ID (Supplementary Table 4). Circular Manhattan plots were visualized using Circos^50^.

### Functionally informed fine-mapping

Functionally informed genetic fine-mapping was performed using PolyFun v.1.0.0 and Sum of Single Effects (SuSiE, v.0.11.92)^51,52^. PolyFun was used to estimate per-SNP heritability using L2-regularized extension of stratified LDSC (S-LDSC) and baseline LD model v.2.2 containing 187 annotations^48,49,51^. The estimated per-SNP heritabilities were used as prior causal probabilities in SuSiE with a maximum of ten causal variants in each region. The subset of 337,000 unrelated white-British individuals from UK Biobank were used for in-sample LD structure. After excluding the MHC region on chromosome 6, fine-mapping per each locus was performed in a window of 1.5 Mb around the lead genetic variants (*P* < 5 × 10^−8^).

### Colocalization

Colocalization was performed between independent genetic loci identified by COJO-GCTA, and summary statistics of gene expression quantitative trait loci (eQTL) of 49 tissues in GTEx (v.8) from the eQTL catalog release 4 (refs ^53,54^). The coordinates of GWAS summary statistics were first converted from Build 37 to 38 using liftOver function of rtracklayer R package (v.1.54.0)^55^. We performed colocalization using COLOC-SuSiE assuming the presence of multiple causal variants (coloc R package^56^, v.5.1.0) with default priors and considered variants in a window of 1.5 Mb around the index variant at each locus. We considered only genes with at least one significant variant (FDR *P* < 0.1, eGenes) and performed the colocalization in a window of 1.5 Mb around each eGene. If SuSiE did not converge after 1,000 iterations, conventional (single causal variant) colocalization was used. Finally, an H4 posterior probability (PP) > 0.8 was considered as strong evidence that both traits share the same causal variant.

### Variant annotation

Independent genome-wide significant and fine-mapped variants were annotated using Ensembl Variant Effect Predictor (VEP) accessed from REST API (https://rest.ensembl.org/).

### Gene mapping and functional enrichment analysis

To map and prioritize potential candidate genes for independent genetic loci, we employed multiple approaches. (1) SNP2GENE module of FUMA v.1.5.4 (ref. ^57^) was used for positional mapping of lead variants to genes with a maximum distance of 50 kb. (2) eQTL mapping using FUMA by considering only genes with at least one significant eQTL association (FDR < 0.05). (3) 3D chromatin interaction mapping using FUMA by considering only significant interactions (FDR < 1 × 10^−6^) within 250–500 bp upstream and downstream of the transcription start site, respectively. (4) Multi-marker analysis of genomic annotation (MAGMA, v.1.08)^58^ implemented in FUMA was carried out to perform genome-wide gene association analysis using 19,535 curated protein-coding genes. Only genes with a Bonferroni threshold below 0.05/19,535 = 2.56 × 10^−6^ were kept for gene mapping. Variants within the MHC region were excluded before the analysis. (5) Colocalized genes from colocalization analysis with at least one tissue and an H4 PP > 0.8. (6) Nearest gene(s) to the fine-mapped variants with the maximum PP per each locus. (7) Genes with the highest overall V2G score at each locus were based on Open Targets Genetics^59^. Finally, to prioritize the mapped genes, we calculated an unweighted ranking score by summing over the evidence from the above-mentioned approaches.

By using the set of genes with the maximum ranking score at each locus, we performed functional gene-set enrichment analysis using Enrichr tool^60^ against, ARCHS4 tissues, Reactome biological pathways and Gene Ontology Biological Processes. Significant terms with a Benjamini–Hochberg FDR-corrected *P* value <0.05 per each database were reported. For visualization, both adjusted *P* values and Enrichr combined scores (−log(*P*) × OR) were used.

### Partitioned polygenic risk scores of liver triglyceride content

To define the pPRSs, genetic loci (full list in Supplementary Table 15) were assigned into two groups based on their concordant or discordant associations with PDFF and circulating triglycerides. We excluded the genetic loci that did not associate with circulating triglycerides. Finally, the pPRSs were generated by taking the weighted sum of genetic variants, where the strongest association at each locus was used as the weight, following the pleiotropy analysis.

### Replication cohorts

#### NEO

NEO is a population-based cohort study in men and women aged 45 to 65 years, with oversampling of individuals having BMI over 27 kg m^−2^ from Leiden and surrounding areas in the Netherlands. At baseline, 6,671 participants were included and around 35% of the NEO participants were randomly selected to undergo hepatic triglyceride content (HTGC) measurements by magnetic resonance spectroscopy. Genotyping was performed using Illumina HumanCoreExome-24 BeadChip and imputed to TOPMed reference genome panel^61^. In the present work, a total of 1,822 individuals of European ancestry with an available HTGC were used.

#### Liver BIBLE

The Liver BIBLE-2022 cohort comprises 1,144 healthy middle-aged individuals (40–65 years) with metabolic dysfunction (at least three criteria for metabolic syndrome among BMI ≥ 35 kg m^−^^2^, arterial hypertension ≥135/80 mm Hg or therapy, fasting glucose ≥100 mg dl^−1^ or diabetes, low high-density lipoprotein <45/55 mg dl^−1^ in males/females and high triglycerides ≥150 mg dl^−1^) who presented for blood donation from June 2019 to February 2021 at the Transfusion Medicine unit of Fondazione IRCCS Ca’ Granda Hospital (Milan, Italy)^62^. Hepatic fat content was estimated noninvasively by controlled attenuation parameter (CAP) with FibroScan device (Echosens). Genotyping was performed by Illumina GlobalScreeningArray (GSA)-24 v.3.0 plus Multidisease Array (Illumina) and further imputed to TOPMed reference genome panel^63^. At the time of analysis, genomic data passing quality control with an available CAP measure were available for 1,081 patients of European ancestry.

#### MAFALDA

The MAFALDA study started in May 2020 and ended in April 2022. It comprised a total of 468 consecutive participants with morbid obesity (BMI ≥ 35 kg m^−^^2^) who underwent bariatric surgery at Campus Bio-Medico University of Rome, Italy. In MAFALDA participants, SLD diagnosis was assessed only by liver histology in *n* = 116, only by vibration-controlled transient elastography, including CAP measurement with FibroScan (Echosens)^64^ in 141 individuals, with both in 148 individuals and 63 with neither CAP nor liver biopsy. In this study, only individuals with liver fat content estimated by CAP were included (*n* = 172). Genotyping was performed in the same manner as that of the Liver BIBLE cohort. MAFALDA includes a total of 264 paired visceral and liver biopsies with available bulk transcriptomic data.

#### Dallas Heart Study

In this study, only 828 European Americans from the Dallas Heart Study (DHS-1) were used. The DHS is a population-based sample study of Dallas County, Texas, USA, where liver triglyceride content was measured by magnetic spectroscopy. Details of this study can be found elsewhere^13^.

#### Ethics

This research complies with the principles outlined in the Declaration of Helsinki. The UK Biobank received ethical approval from the National Research Ethics Service Committee Northwest Multi-Centre Haydock (reference 16/NW/0274). Data used in this study were obtained under application number 37142. The NEO study was approved by the medical ethical committee of the Leiden University Medical Center. The Liver BIBLE study was approved by the ethical committee of the Fondazione IRCCS Ca’ Granda (ID 1650, revision 23 June 2020). The MAFALDA study was approved by the Local Research Ethics Committee (no. 16/20). The DHS was approved by the institutional review board of the University of Texas Southwestern Medical Center. Each participant provided written informed consent. The baseline characteristics for these cohorts are listed in Supplementary Table 23.

### Meta-analysis

The association between previously unknown independent loci for PDFF and cT1 and magnetic resonance spectroscopy liver fat (DHS-1 and NEO studies) or CAP measurement (MAFALDA and Liver BIBLE) was performed using a linear regression analysis adjusted for age, sex, age^2^, age × sex, age^2^ × sex and BMI after a rank-based inverse normal transformation of the response. An inverse-variance meta-analysis was then performed with fixed-effect model using the meta R package (v.6.5.0). For genetic variants not available in either of replication cohorts, a proxy variant was used instead: variants in LD (*R*^2 ^> 0.4) with the lead variant in the UK Biobank within a window of 1.5 Mbp. If no such variant was found in the UK Biobank, the LDproxy tool with Europeans from 1000 Genomes Project was used instead^20^. In case of multiple proxy variants, the one with the highest LD and functional consequence was selected.

### RNA-seq analysis

Total RNA for 264 paired liver and VAT samples from the MAFALDA cohort was isolated using miRNeasy Advanced Mini kit (QIAGEN). RNA sequencing and library preparation was performed in a paired-end 150-bp mode using the Illumina NovaSeq PE150 (Novogene). Reads were aligned to GRCh38 reference genome by STAR^65^ (v.2.7.10a) after quality check (FastQC software v.0.12.0, Babraham Bioinformatics) and trimming of low-quality reads and potential contaminating adapters by Trimmomatic^66^ (v.0.39). Gene-level read counts were quantified by RSEM^67^ (v.1.3.3) tool against the Ensembl (release 107). Samples with insufficient mapping specificity (uniquely to total mapped reads <0.7) were excluded before the analysis. Finally, a paired differential expression analysis of 261 VAT and 244 liver samples was carried out using DESeq2 (ref. ^68^) (v.1.38.3), while adjusting for RNA integrity number, individual ID and five surrogate variables detected by surrogate variable analysis^69^.

### Follow-up analysis

The longitudinal association of PRS with the occurrence of the outcomes was tested through Cox proportional hazard regression and expressed as hazard ratios with 95% confidence intervals. The median follow-up was 14.5 years and individuals with any of diagnoses at the baseline were excluded before the analysis (Supplementary Table 22). The proportional hazard assumption was checked through the consideration of Schoenfeld residuals and no violations were detected. Prospective associations were performed in R v.4.0.2 (R Foundation for Statistical Computing).

### Gene–adiposity interaction analysis

Gene–adiposity interaction for independent loci from multi-adiposity-adjusted GWAS was performed in REGENIE (v.3.2.8) using robust standard errors (sandwich estimators HC3) to guard against heteroskedasticity^16^. The analysis was adjusted for age at MRI, sex, age^2^, age × sex, age^2^ × sex, the first ten PCs of ancestry, genotyping array and adiposity index, where adiposity index was VAT, WFM or BMI. Due to the sensitivity of interaction effect sizes to the trait transformation, PDFF and liver iron corrected T1 were log-transformed before the interaction analyses^70,71^.

### Mediation analysis

To examine whether the impact of identified independent loci on PDFF or liver iron corrected T1 are mediated via the measures of adiposity, we performed mediation analysis using the mediation R package^72^. All models were adjusted for age at MRI, sex, age^2^, age × sex, age^2^ × sex, the first ten PCs of ancestry, genotyping array and the polygenic covariate estimated in step 1 of REGENIE^16^. We additionally considered the scenario with a genetic variant-mediator interaction term. The significance of mediation (*P* values and 95% CIs) was assessed via a nonparametric bootstrap method (1,000 simulations) on the rank-based inverse normal transformed PDFF and liver iron corrected T1 as outcomes and adiposity measures as mediators. We also performed sensitivity analyses to examine sequential ignorability assumption (possible existence of unobserved confounders between adiposity indices and liver traits)^30^. This was carried out by examining the correlation coefficient between error terms of liver traits and adiposity index models, at which estimated mediation effect was zero (95% CI contains 0).

### Association analysis with adiposity measures

The association between independent loci from multi-adiposity-adjusted GWAS and BMI, WFM or VAT was performed using a whole-genome regression model as implemented in REGENIE (v.3.2.8)^16^. All models were adjusted for age at MRI, sex, age^2^, age × sex, age^2^ × sex, the first ten PCs of ancestry and genotyping array. Measures of adiposity were rank-based inverse normal transformed before the analysis.

### bNMF clustering

We applied bNMF, an unsupervised soft clustering approach, to define ‘hypothesis-free’ clusters of independent loci from multi-adiposity-adjusted GWAS^35^. This approach has been successfully employed to find physiologically relevant partitioned polygenic risk scores for type 2 diabetes^35^. We first performed the association analysis between ALT, aspartate aminotransferase, glycated hemoglobin, circulating triglycerides, low-density lipoprotein cholesterol, glucose, creatinine, systolic blood pressure and cystatin C using REGENIE adjusted for age, sex, age^2^, age × sex, age^2^ × sex, the first ten PCs of ancestry and adiposity index, where adiposity index was chosen based on the strongest association from the multi-adiposity-adjusted GWAS. As VAT was not available at baseline, we used WHR instead. Next, a variant-trait association matrix of standardized *z*-scores (*m* × *n*, where *m* and *n* are the number of independent loci associating with PDFF and continuous traits mentioned above, respectively) was constructed while accounting for different sample sizes in each GWAS on continuous traits^35^. This scaled matrix was then aligned to PDFF-increasing alleles. bNMF clustering was performed using the bNMF R pipeline (https://github.com/gwas-partitioning/bnmf-clustering) by setting the maximum number of clusters, *K*, to 7 for 1,000 iterations and removing highly correlated traits (Pearson correlation coefficient >0.85). After determining the maximum posterior solution at the most probable *K*, a cutoff maximizing the signal-to-noise ratio (1.08) was used to keep variants in each cluster^35^. Two top-weighted traits at each cluster were used to define the cluster names. Finally, pPRSs were generated by a weighted sum of genetic variants at each cluster, where weights were derived from the multi-adiposity-adjusted GWAS of PDFF.

### Comparison between bNMF and PDFF-TGs pPRS

To compare the PDFF-TGs hypothesis-driven pPRS approach and two pPRSs identified by bNMF algorithm to distinguish between liver, cardiometabolic and kidney outcomes, we performed a Wald test as follows:

$$W=\frac{{\hat{\beta }}_{{pPRS}1}\,-\,{\hat{\beta }}_{{pPRS}2}}{\sqrt{{{SE}}_{{pPRS}1}^{2}+\,{{SE}}_{{pPRS}2}^{2}}}$$

where ${\hat{\beta }}_{{pPRS}1}$, ${{{{SE}}_{{pPRS}1}^{2}}}$ and ${\hat{\beta }}_{{pPRS}2}$, ${{{{SE}}_{{pPRS}2}^{2}}}$ are the log OR and standard error of either discordant/concordant or bNMF pPRSs, respectively. In the equation above, *W* is the Wald test statistic and ${W}^{2} \sim \,{\chi }_{1}^{2}$. Similarly, we compared the model fit, by calculating the difference between Akaike information criterion between the pPRS (Supplementary Table 19).

### Reporting summary

Further information on research design is available in the Nature Portfolio Reporting Summary linked to this article.

## Online content

Any methods, additional references, Nature Portfolio reporting summaries, source data, extended data, supplementary information, acknowledgements, peer review information; details of author contributions and competing interests; and statements of data and code availability are available at 10.1038/s41591-024-03284-0.

## Supplementary information

Supplementary Information Supplementary Figs. 1–6.

Reporting Summary

**Supplementary Table 1** Characteristics of European participants from UK Biobank with available PDFF or liver iron corrected T1 (cT1) stratified by sex. **Supplementary Table 2** Heritability estimates of PDFF and liver iron corrected T1 under different adiposity adjustments. **Supplementary Table 3** Genetic correlation among PDFF and liver iron corrected T1 under different adiposity adjustments. **Supplementary Table 4** Pleiotropy analysis of PDFF and liver iron corrected T1 under different adiposity adjustments. **Supplementary Table 5** Multi-adipo-adjusted GWAS of PDFF and cT1 across different adjustments for adiposity indices in the UK Biobank. **Supplementary Table 6a** Overlapping between previously reported genomic loci GWAS catalog and independent loci found in present study for PDFF. **Supplementary Table 6b** Overlapping between previously reported genomic loci GWAS catalog and independent loci found in present study for liver iron corrected T1. **Supplementary Table 7a** Functionally informed fine-mapping results for PDFF. **Supplementary Table 7b** Functionally informed fine-mapping results for liver iron corrected T1. **Supplementary Table 8a** Colocalization with GTEx eQTLs for PDFF. **Supplementary Table 8b** Colocalization with GTEx eQTLs for liver iron corrected T1. **Supplementary Table 9a** Gene mapping for independent genetic loci for PDFF. **Supplementary Table 9b** Gene mapping for independent genetic loci for liver iron corrected T1. **Supplementary Table 10a** Gene-set enrichment analysis of mapped genes from PDFF independent genetic loci*.* **Supplementary Table 10b** Gene-set enrichment analysis of mapped genes from liver iron corrected T1 independent genetic loci. **Supplementary Table 10c** Gene-set enrichment analysis of mapped genes for independent genetic loci in PDFF-circulating TGs discordant pPRS. **Supplementary Table 10d** Gene-set enrichment analysis of mapped genes for independent genetic loci in PDFF-circulating TGs concordant pPRS. **Supplementary Table 11** The association between independent novel genetic loci and liver and metabolic-related outcomes in the UK Biobank. **Supplementary Table 12** Summarized results of mediation, interaction and association analyses for PDFF and cT1 across different measures of adiposity. **Supplementary Table 13** Summary statistics of mediation, interaction and association analyses for PDFF and cT1 across different measures of adiposity. **Supplementary Table 14** Meta-analysis of the associations between independent novel genetic loci and HTGC in four independent cohorts. **Supplementary Table 15** The association between independent previously known and replicated novel genetic loci from adipo-adjusted PDFF and liver iron corrected T1 and circulating triglycerides in the UK Biobank. **Supplementary Table 16** Goodness-of-fit for pPRS. **Supplementary Table 17** The association between genetic variants used in PDFF-circulating TGs pPRS and liver-related, cardiometabolic and chronic kidney failure traits in the UK Biobank. **Supplementary Table 18** Case–control and prospective association of discordant and concordant pPRS with liver-related, cardiometabolic and chronic kidney failure traits in the UK Biobank. **Supplementary Table 19** Comparison of PDFF-TGs and bNMF clustering pPRS approaches. **Supplementary Table 20** Baseline characteristics of individuals stratified by the extremes of pPRS. **Supplementary Table 21** Sex-specific association of discordant and concordant pPRS with liver-related, cardiometabolic and chronic kidney failure traits in the UK Biobank. **Supplementary Table 22** Definition of binary traits. **Supplementary Table 23** Baseline characteristics of European participants in replication cohorts stratified by sex.

## Data Availability

All data associated with this study are presented in the paper or the Supplementary Information. Multi-adiposity-adjusted GWAS of PDFF and liver iron corrected T1 (GRCh37) are publicly available on GWAS catalog with the following accession IDs: GCST90446646, GCST90446647, GCST90446648, GCST90446649, GCST90446650, GCST90446651, GCST90446652 and GCST90446653. All external GWAS summary statistics accessed via the GWAS catalog are publicly available and have been cited in Supplementary Table 6a,b. For the UK Biobank, all individual-level phenotype/genotype data are accessible via a formal application to the UK Biobank at http://www.ukbiobank.ac.uk. The ethical approval of the MAFALDA study restricts the public sharing of individual data; however, the data of the liver visceral adipose biopsies from the MAFALDA cohort researchers can submit a proposal to access either raw or analyzed data between 9 to 36 months after publication. Proposals should be directed to S.R. at stefano.romeo@ki.se. S.R. will review each request to assess data availability. Responses will be provided within 8 weeks of receiving the request. It is important to note that patient-related data may be restricted due to confidentiality regulations. If approved for sharing, data will be transferred under a material transfer agreement. NEO study requests should be sent to f.r.rosendaal@lumc.nl. Liver BIBLE study requests should be sent to luca.valenti@unimi.it. Dallas Heart Study requests should be sent to dallasheartstudy@utsouthwestern.edu. The following online databases have been used: GWAS catalog, https://www.ebi.ac.uk/gwas/ and baseline LD model: https://data.broadinstitute.org/alkesgroup/LDSCORE/.

## Code availability

All codes and scripts used for analyses are available at https://github.com/Ojami/PartiotionedPRS_custom. MATLAB R2023a was used under an academic license. Publicly available tools used in this work are listed as follows: REGENIE v.3.2.8 (https://github.com/rgcgithub/regenie); PLINK v.1.90b6.26 (https://www.cog-genomics.org/plink/); GCTA-COJO (https://yanglab.westlake.edu.cn/software/gcta/#COJO); LDSC (https://github.com/bulik/ldsc/); Circos (http://circos.ca/); PolyFun: https://github.com/omerwe/polyfun); SuSiE v.0.11.92 (https://github.com/stephenslab/susieR); rtracklayer v.1.54.0 (https://bioconductor.org/packages/release/bioc/html/rtracklayer.html); coloc v.5.1.0 (https://github.com/chr1swallace/coloc); Ensembl VEP REST API (https://rest.ensembl.org/); FUMA v.1.5.4 and MAGMA (https://fuma.ctglab.nl/); Open Targets Genetics (https://genetics.opentargets.org); Enrichr R wrapper (https://github.com/wjawaid/enrichR); meta (https://cran.r-project.org/web/packages/meta/index.html); LDproxy (https://ldlink.nih.gov/?tab=ldproxy); STAR v.2.7.10a (https://github.com/alexdobin/STAR); FastQC (https://www.bioinformatics.babraham.ac.uk/projects/fastqc/); Trimmomatic v.0.39 (http://www.usadellab.org/cms/?page=trimmomatic); RSEM v.1.3.3 (https://github.com/deweylab/RSEM); DESeq2 v.1.38.3 (https://bioconductor.org/packages/release/bioc/html/DESeq2.html); R v4.0.2 (https://www.r-project.org/); bNMF (https://github.com/gwas-partitioning/bnmf-clustering); and the mediation R package v.4.5.0 (https://cran.r-project.org/web/packages/mediation/index.html).
