# Publisher Correction: Partitioned polygenic risk scores identify distinct types of metabolic dysfunction-associated steatotic liver disease

**DOI:** 10.1038/s41591-025-03503-2

**Published:** 2025-01-16

**Authors:** Oveis Jamialahmadi, Antonio De Vincentis, Federica Tavaglione, Francesco Malvestiti, Ruifang Li-Gao, Rosellina M. Mancina, Marcus Alvarez, Kyla Gelev, Samantha Maurotti, Umberto Vespasiani-Gentilucci, Frits Richard Rosendaal, Julia Kozlitina, Päivi Pajukanta, François Pattou, Luca Valenti, Stefano Romeo

**Affiliations:** 1 Department of Molecular and Clinical Medicine, Institute of Medicine, Sahlgrenska Academy, Wallenberg Laboratory, University of Gothenburg, Gothenburg, Sweden; 2 Operative Unit of Internal Medicine, Fondazione Policlinico Universitario Campus Bio-Medico, Rome, Italy; 3 Research Unit of Internal Medicine, Department of Medicine and Surgery, Università Campus Bio-Medico di Roma, Rome, Italy; 4 Operative Unit of Clinical Medicine and Hepatology, Fondazione Policlinico Universitario Campus Bio-Medico, Rome, Italy; 5 Research Unit of Clinical Medicine and Hepatology, Department of Medicine and Surgery, Università Campus Bio-Medico di Roma, Rome, Italy; 6 Department of Pathophysiology and Transplantation, Università degli Studi di Milano, Milan, Italy; 7 Department of Clinical Epidemiology, Leiden University Medical Center, Leiden, the Netherlands; 8 Department of Life Science, Health, and Health Professions, Link Campus University, Rome, Italy; 9 Department of Human Genetics, David Geffen School of Medicine at UCLA, Los Angeles, CA USA; 10 Department of Experimental and Clinical Medicine, Magna Graecia University, Catanzaro, Italy; 11 The Eugene McDermott Center for Human Growth and Development, University of Texas Southwestern Medical Center, Dallas, TX USA; 12 Bioinformatics Interdepartmental Program, UCLA, Los Angeles, CA USA; 13 Institute for Precision Health, David Geffen School of Medicine at UCLA, Los Angeles, CA USA; 14 Service de chirurgie générale et endocrinienne, Centre Hospitalier Universitaire de Lille, Lille, France; 15 European Genomic Institute for Diabetes, UMR 1190 Translational Research for Diabetes, Inserm, CHU Lille, University of Lille, Lille, France; 16 Precision Medicine - Biological Resource Center, Department of Transfusion Medicine, Fondazione IRCCS Ca’ Granda Ospedale Maggiore Policlinico, Milan, Italy; 17 Department of Cardiology, Sahlgrenska University Hospital, Gothenburg, Sweden; 18 Clinical Nutrition Unit, Department of Medical and Surgical Sciences, University Magna Graecia, Catanzaro, Italy; 19 Department of Medicine (H7), Karolinska Institute, Huddinge, Stockholm, Sweden; 20 Department of Endocrinology, Karolinska University Hospital, Huddinge, Stockholm, Sweden

**Keywords:** Risk factors, Genome-wide association studies

Correction to: *Nature Medicine* 10.1038/s41591-024-03284-0, published online 9 December 2024.

In the version of the article initially published, the reference Raverdy, V. et al. Data-driven cluster analysis identifies distinct types of metabolic dysfunction-associated steatotic liver disease. *Nat. Med*. 10.1038/s41591-024-03283-1 (2024) was missing and has now been added to the tenth paragraph of the Discussion, in the sentence beginning “Finally, we obtained similar results by using a completely different approach, namely, unsupervised clustering…”. This correction has been made to the HTML and PDF versions of the article.

