# Supplementary material for: Partitioned polygenic risk scores identify distinct types of metabolic dysfunction-associated steatotic liver disease
Source: Nat Med. 2024 Dec 9;30(12):3614–23. doi: 10.1038/s41591-024-03284-0 (PMC11645285; doi:10.1038/s41591-024-03284-0)
Supplement: Supplementary file 1 — Supplementary Figs. 1–6. [file 41591_2024_3284_MOESM1_ESM.pdf]

nature medicine

Supplementary information https://doi.org/10.1038/s41591-024-03284-0

Partitioned polygenic risk scores identify distinct types of metabolic dysfunction-associated steatotic liver disease

In the format provided by the authors and unedited

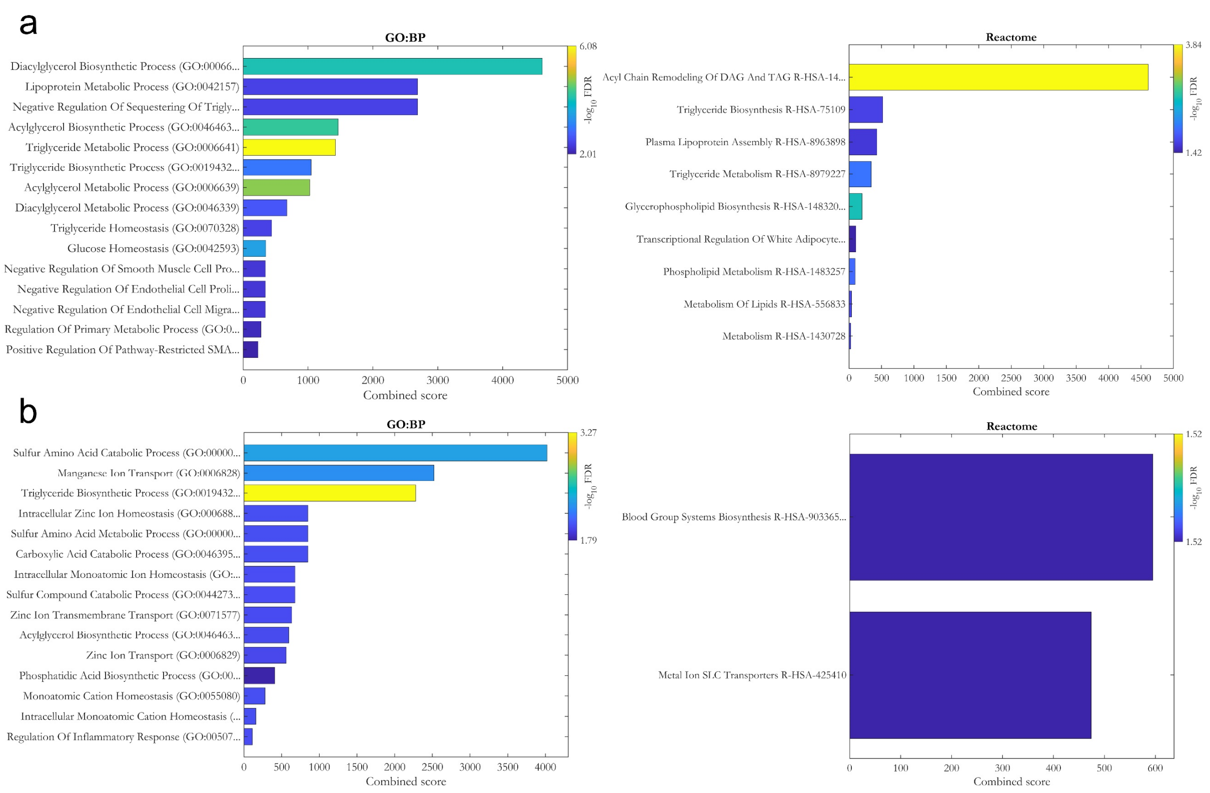

Supplementary Figure 1. Gene-set enrichment analysis of mapped genes from PDFF and cT1 independent genetic *loci*. Mapped-genes with the highest evidence rank at each locus for (a) PDFF and (b) cT1 were used to perform functional gene-set enrichment analysis. X-axis shows combined score calculated by Enrichr tool as -log(p-value) ×odds ratio. P-values were calculated by two-sided Fisher's exact test and corrected for Benjamini–Hochberg false discovery rate. PDFF: proton density fat fraction; cT1: liver iron corrected T1. Full summary statistics have been reported in Supplementary Tables 10A and B.

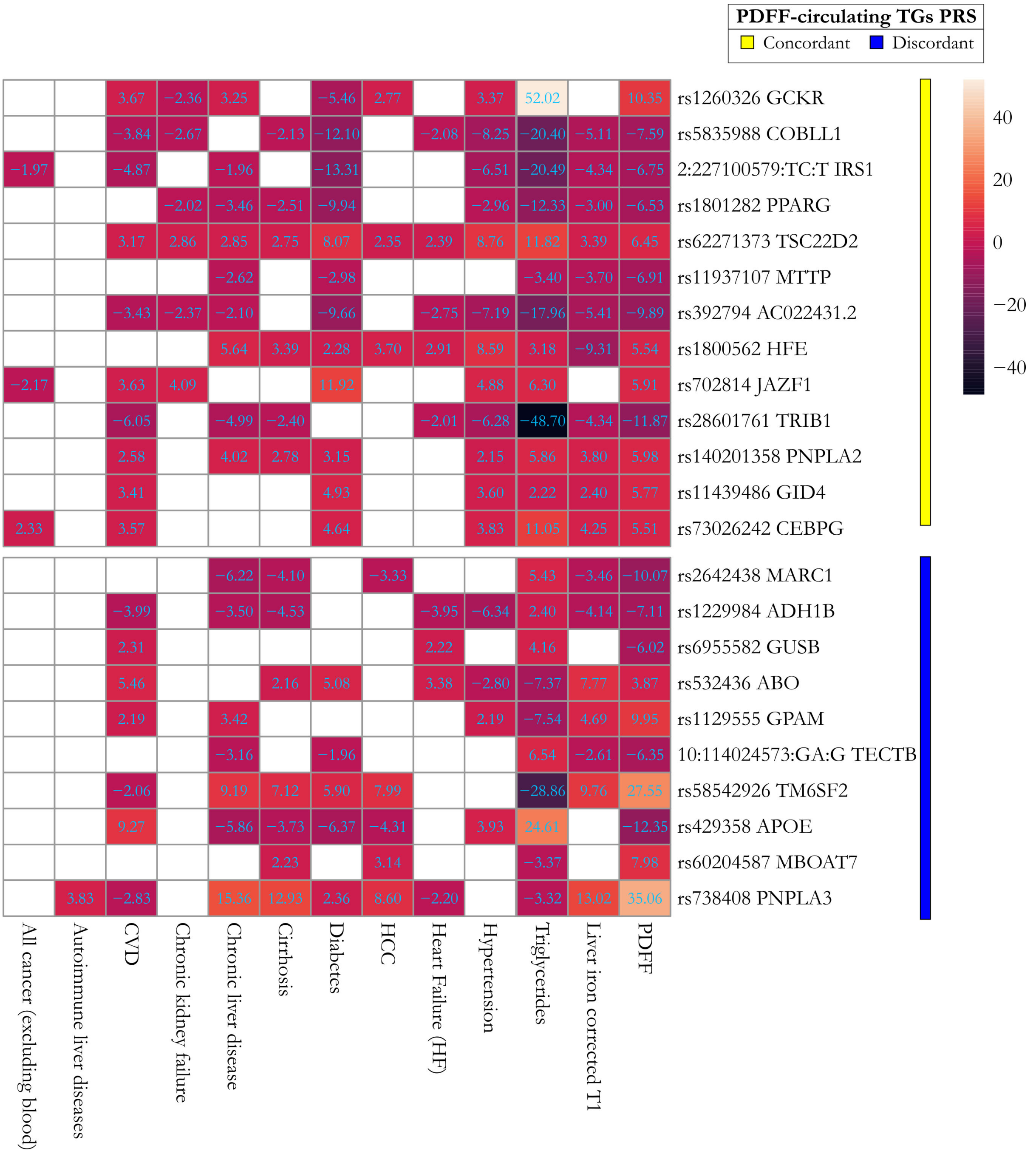

Supplementary Figure 2. Association of variants composing concordant and discordant PRS with liver-related, cardiometabolic, and chronic kidney failure traits in the UK Biobank. Heatmap shows the Z-score of associations between genetic variants used in 2 PRS and each disease (corresponding to Figure 6A). Full summary statistics have been reported in Supplementary Table 17. All association analyses have been performed after excluding individuals with available PDFF using performed by linear or logistic regression analysis using REGENIE and adjusted for BMI, age, sex, age×sex, age^2 and age^2×sex, first 10 genomic principal components and array batch. P-values were two-sided and not corrected for multiple hypothesis testing. TG: triglycerides; PDFF: proton density fat fraction; HCC: hepatocellular carcinoma; CVD: cardiovascular disease; PRS: polygenic risk scores.

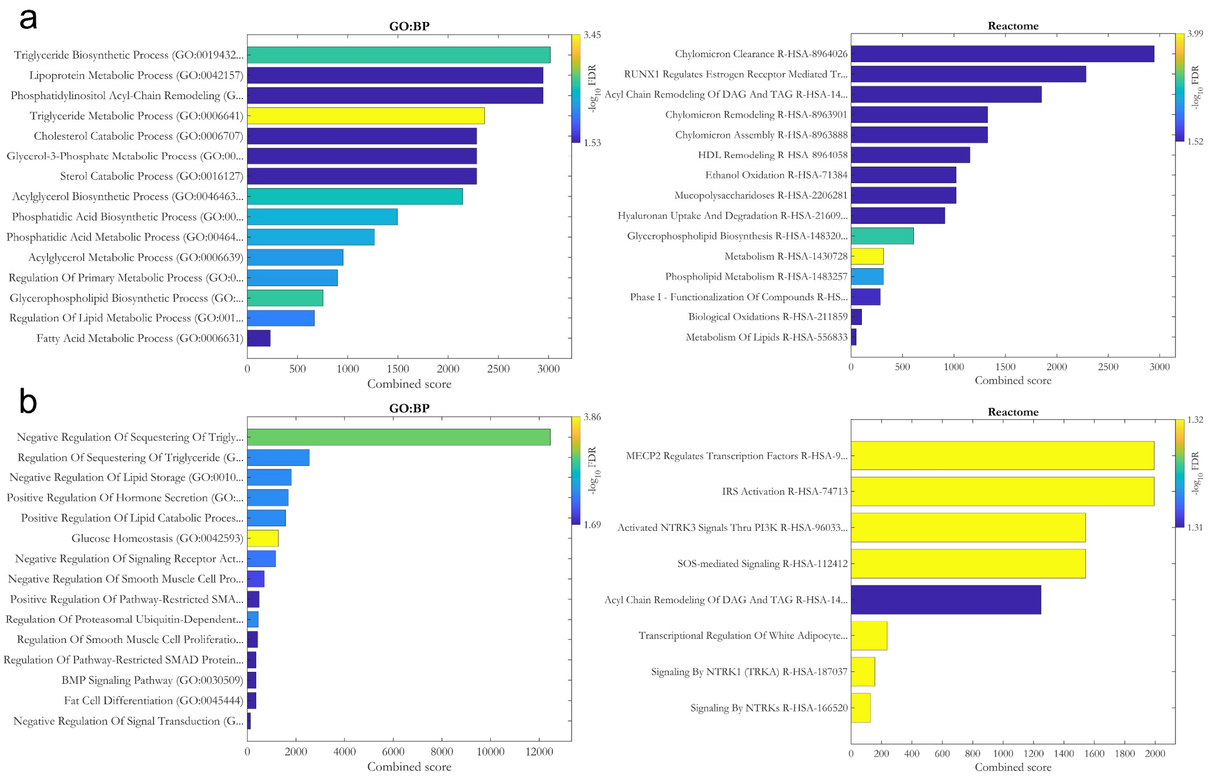

Supplementary Figure 3. Gene-set enrichment analysis of mapped genes from PDFF-circulating TGs PRS. Mapped-genes with the highest evidence rank at each *locus* for (a) discordant and (b) concordant polygenic risk scores (PRS) were used to perform functional gene-set enrichment analysis. X-axis shows combined score calculated by Enrichr tool as -log(p-value) ×odds ratio. P-values were calculated by two-sided Fisher's exact test and corrected for Benjamini–Hochberg false discovery rate. Full summary statistics have been reported in Supplementary Tables 10C and D. TG: triglyceride.

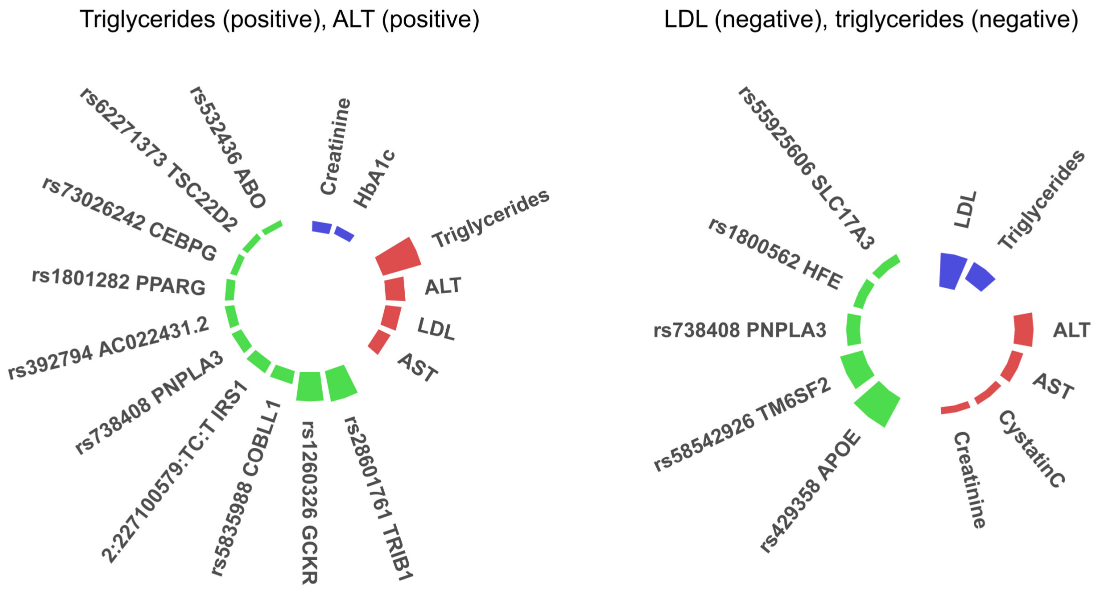

Supplementary Figure 4. Top-weighted genetic-trait clusters of PDFF in the UK Biobank. Circular plots show the two genetic clusters identified by bNMF algorithm. Bar length denotes the cluster weight from bNMF algorithm. Green, red, and blue bars in each cluster denote genetic *loci*, traits with increased and decreased values, respectively. AST: aspartate transaminase, HbA1c: Glycated haemoglobin.

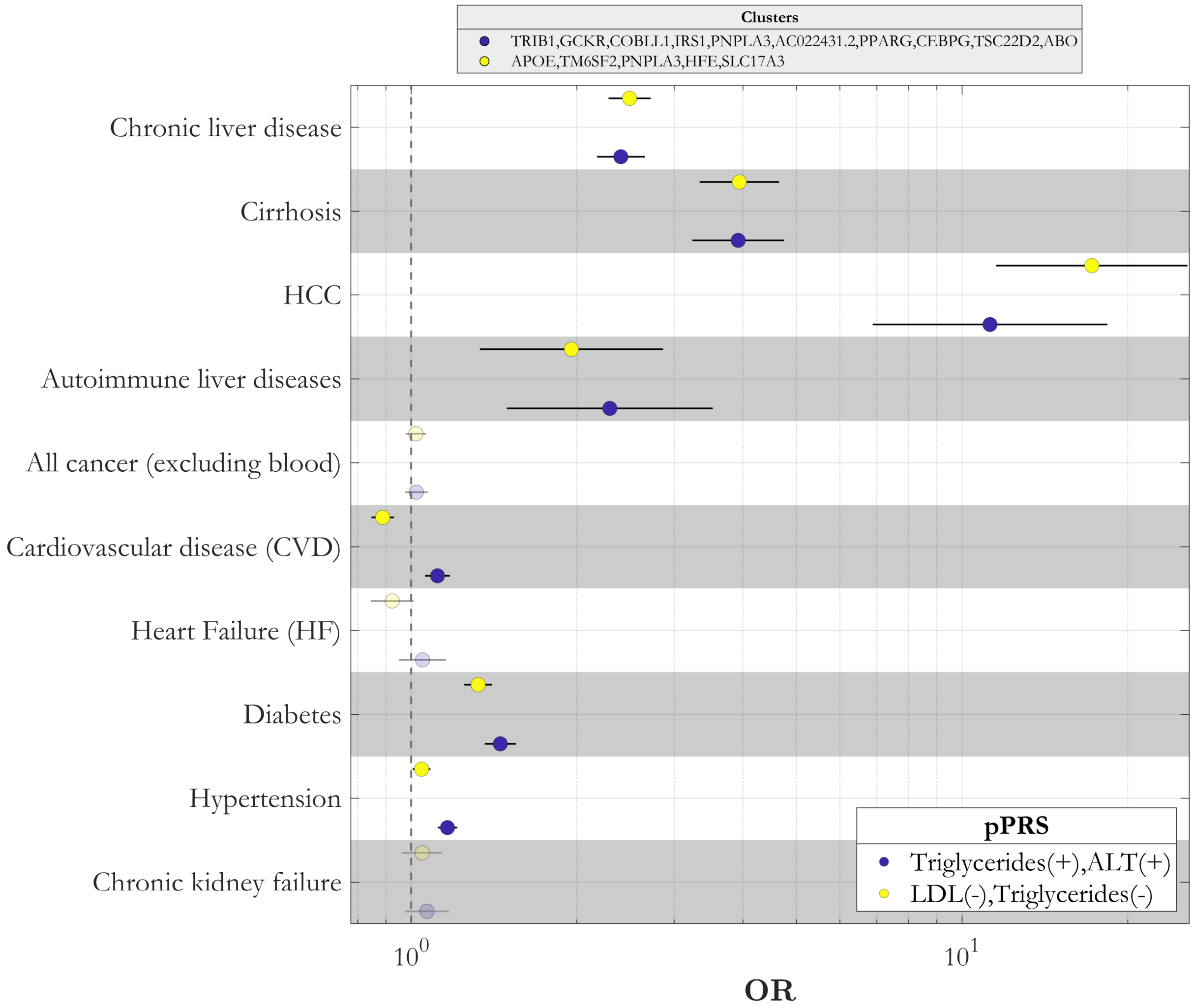

Supplementary Figure 5. Association between 2 bNMF pPRS and liver-related, cardiometabolic, and chronic kidney failure in the UK Biobank. The association between normalized pPRS and each disease was tested using logistic regression analysis adjusted for BMI, age, sex, age×sex, age^2 and age^2×sex, first 10 genomic principal components and array batch. X-axis shows the odds ratio (OR). All association analyses have been performed after excluding individuals with available PDFF (n= 36,394). P-values were two-sided and not corrected for multiple hypothesis testing. pPRS were named using the two top-weighted traits in each cluster. Error bars represent the 95% confidence intervals from the regression models. The variants used in pPRS have been shown in the top legend. TG: triglyceride; PDFF: proton density fat fraction; HCC: hepatocellular carcinoma; pPRS: partitioned polygenic risk scores.

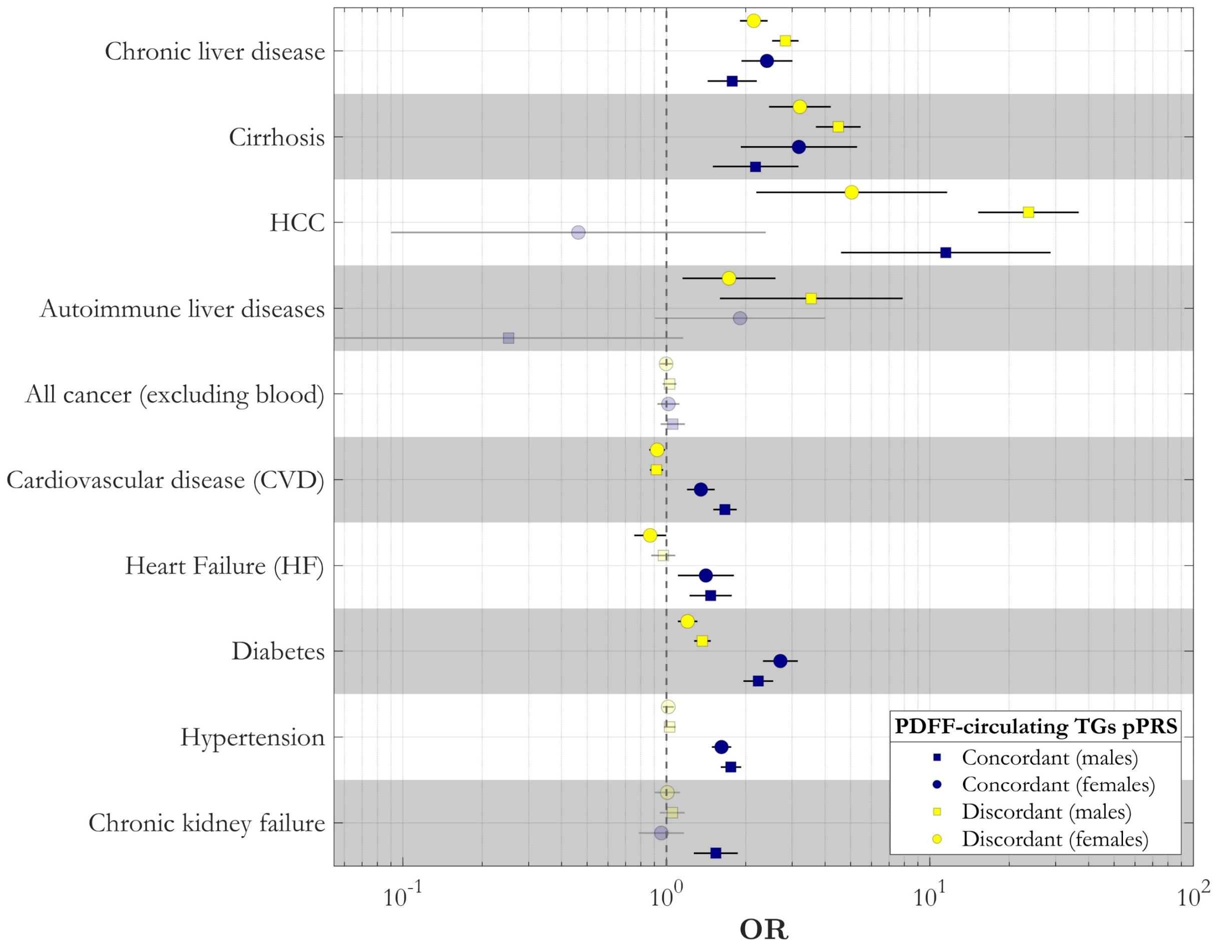

Supplementary Figure 6. Sex-specific association of discordant and concordant pPRS with liver-related, cardiometabolic, and chronic kidney failure traits in the UK Biobank. The association between concordant and discordant PDFF-circulating triglyceride levels PRS with each disease was tested using logistic regression analysis adjusted for BMI, age, age^2, first 10 genomic principal components and array batch. X-axis shows either the odds ratio (OR). All association analyses have been performed after excluding individuals with available PDFF (n= 36,394). Error bars represent the 95% confidence intervals from the regression models. Full summary statistics have been reported in Supplementary Table 21. P-values were two-sided and not corrected for multiple hypothesis testing. TG: triglyceride; PDFF: proton density fat fraction; HCC: hepatocellular carcinoma; pPRS: partitioned polygenic risk scores.
