# Supplementary material for: Partitioned polygenic risk scores identify distinct types of metabolic dysfunction-associated steatotic liver disease
Source: Nat Med. 2024 Dec 9;30(12):3614–23. doi: 10.1038/s41591-024-03284-0 (PMC11645285; doi:10.1038/s41591-024-03284-0)
Supplement: Supplementary file 2 — Reporting Summary [file 41591_2024_3284_MOESM2_ESM.pdf]

nature portfolio

Corresponding author(s): Oveis Jamialahmadi
Stefano Romeo

Last updated by author(s): Aug 27, 2024

Reporting Summary

Nature Portfolio wishes to improve the reproducibility of the work that we publish. This form provides structure for consistency and transparency in reporting. For further information on Nature Portfolio policies, see our Editorial Policies and the Editorial Policy Checklist.

Statistics

For all statistical analyses, confirm that the following items are present in the figure legend, table legend, main text, or Methods section.

n/a	Confirmed	
☐	☒	The exact sample size (*n*) for each experimental group/condition, given as a discrete number and unit of measurement
☒	☐	A statement on whether measurements were taken from distinct samples or whether the same sample was measured repeatedly
☐	☒	The statistical test(s) used AND whether they are one- or two-sided<br>*Only common tests should be described solely by name; describe more complex techniques in the Methods section.*
☐	☒	A description of all covariates tested
☐	☒	A description of any assumptions or corrections, such as tests of normality and adjustment for multiple comparisons
☐	☒	A full description of the statistical parameters including central tendency (e.g. means) or other basic estimates (e.g. regression coefficient) AND variation (e.g. standard deviation) or associated estimates of uncertainty (e.g. confidence intervals)
☐	☒	For null hypothesis testing, the test statistic (e.g. *F*, *t*, *r*) with confidence intervals, effect sizes, degrees of freedom and *P* value noted<br>*Give P values as exact values whenever suitable.*
☐	☒	For Bayesian analysis, information on the choice of priors and Markov chain Monte Carlo settings
☒	☐	For hierarchical and complex designs, identification of the appropriate level for tests and full reporting of outcomes
☐	☒	Estimates of effect sizes (e.g. Cohen's *d*, Pearson's *r*), indicating how they were calculated

Our web collection on statistics for biologists contains articles on many of the points above.

Software and code

Policy information about availability of computer code

Data collection	No software was used for data collection.
Data analysis	All codes and scripts used for analyses are available at https://github.com/Ojami/PartiotionedPRS_custom. Penalized regression model selection and correlation coefficients' calculations were performed in MATLAB R2023a: https://www.mathworks.com/. Genome-wide association studies were performed using REGENIE v3.2.8: https://github.com/rgcgithub/regenie. LD clumping and variant filtering was performed using PLINK v1.90b6.26: https://www.cog-genomics.org/plink/. Statistical independent variants were identified using GCTA-COJO v.1.94.0: https://yanglab.westlake.edu.cn/software/gcta/#COJO. SNP-heritability and pairwise genetic correlations were estimated using LD score regression analysis (LDSC) v1.0.1: https://github.com/bulik/ldsc/. Circular Manhattan plots were drawn using Circos: http://circos.ca/. For fine-mapping, PolyFun Python package v1.0.0 (https://github.com/omerwe/polyfun) along with SuSiE v0.11.92 (https://github.com/stephenslab/susieR) were used to estimate posterior inclusion probabilities. For colocalization, after lifting over variants from Build 37 to 38 using rtracklayer R package v.1.54.0 (https://bioconductor.org/packages/release/bioc/html/rtracklayer.html), COLOC-SuSiE approach of coloc R package v.5.1.0 (https://github.com/chr1swallace/coloc) was used. Ensembl Variant Effect Predictor (VEP) REST API (https://rest.ensembl.org/) was used for variant annotation. Gene mapping using FUMA v1.5.4 along with gene-based association study of common variants implemented in MAGMA v1.08, were performed on FUMA web server: https://fuma.ctglab.nl/. V2G scores for each variant were extracted from the Open Targets Genetics portal at https://genetics.opentargets.org. Functional gene-set enrichment analysis was conducted using the R wrapper for Enrichr tool v3.2 (https://github.com/wjawaid/enrichR). An inverse-variance-weighted fixed-effect meta-analysis on the summary statistics from the replication cohorts was performed using meta R package v6.5.0 (https://cran.r-project.org/web/packages/meta/index.html). For variants not available in the replication cohorts, proxy variants were identified using LDproxy (https://ldlink.nih.gov/?tab=ldproxy). Bulk RNA-seq analysis and quality control of paired liver and visceral adipose tissues were performed using STAR v2.7.10a (https://github.com/alexdobin/STAR), FastQC v0.12.0 (https://www.bioinformatics.babraham.ac.uk/projects/fastqc/), Trimmomatic v0.39 (http://www.usadellab.org/cms/?page=trimmomatic), RSEM v1.3.3 (https://github.com/deweylab/RSEM), and differential expression analysis

was conducted using DESeq2 R package v.1.38.3 (https://bioconductor.org/packages/release/bioc/html/DESeq2.html). Prospective association studies were performed in R v4.0.2 (https://www.r-project.org/). Bayesian nonnegative matrix factorization (bNMF) was performed using bNMF R pipeline (https://github.com/gwas-partitioning/bnmf-clustering). Mediation analysese were performed using mediation R package v4.5.0: https://cran.r-project.org/web/packages/mediation/index.html

For manuscripts utilizing custom algorithms or software that are central to the research but not yet described in published literature, software must be made available to editors and reviewers. We strongly encourage code deposition in a community repository (e.g. GitHub). See the Nature Portfolio guidelines for submitting code & software for further information.

Data

Policy information about availability of data

All manuscripts must include a data availability statement. This statement should provide the following information, where applicable:

- Accession codes, unique identifiers, or web links for publicly available datasets
- A description of any restrictions on data availability
- For clinical datasets or third party data, please ensure that the statement adheres to our policy

All data associated with this study are present in the paper or the Supplementary Information. All external GWAS summary statistics accessed via GWAS catalog are publicly available and have been cited in Supplementary Tables 6A and 6B. For UK Biobank, all individual-level phenotype/genotype data are accessible via a formal application to the UK Biobank http://www.ukbiobank.ac.uk. The ethical approval of the MAFALDA study restricts the public sharing of individual data. However, the data of the liver visceral adipose biopsies from the MAFALDA cohort researchers can submit a proposal to access either raw or analyzed data between 9 to 36 months after publication. Proposals should be directed to Stefano Romeo at stefano.romeo@ki.se. Stefano Romeo will review each request to assess data availability. Responses will be provided within 8 weeks of receiving the request. It's important to note that patient-related data may be restricted due to confidentiality regulations. If approved for sharing, data will be transferred under a material transfer agreement. For NEO study requests should be sent to f.r.rosendaal@lumc.nl. For Liver-BIBLE study requests should be sent to luca.valenti@unimi.it. For the Dallas Heart Study requests should be sent to: dallasheartstudy@utsouthwestern.edu. The following online databases have been used: GWAS catalog, https://www.ebi.ac.uk/gwas/; baseline LD model: https://data.broadinstitute.org/alkesgroup/LDSCORE/.

Research involving human participants, their data, or biological material

Policy information about studies with human participants or human data. See also policy information about sex, gender (identity/presentation), and sexual orientation and race, ethnicity and racism.

Reporting on sex and gender	In this study, sex was determined based on central registry at recruitment (from NHS) and self-reporting (https://biobank.ndph.ox.ac.uk/showcase/field.cgi?id=31). All our analyses were adjusted for sex. Thus, our findings are independent from sex and apply to both sexes.
Reporting on race, ethnicity, or other socially relevant groupings	In this study, we used the European subset of UK Biobank individuals by including the individuals self-reported as “Irish” or “any other White background”, and further removed outliers based on first 6 genetic principal components of ancestry. These individuals along with the subset of participants of White British ancestry were considered as Europeans. Participants from replication cohorts were self-reported as European ancestry (MAFALDA and Liver-BIBLE from Italy, NEO study from Netherlands and DHS from European Americans from Dallas, USA). Confounding variables were accounted for by adjusting the regression models for age, sex, indices of adiposity, first 10 genomic principal components and array batch. The confounding bias in the GWA studies were further evaluated using LDSC software (https://github.com/bulik/ldsc/).
Population characteristics	Age; sex; body mass index; genotyping; adiposity measures directly provided by UK Biobank, including visceral adipose tissue volume (VAT, data-field 22407), whole body fat mass (WFM, data-field 23100), impedance of whole body (IWB, data-field 23106); waist-to-hip ratio (WHR) calculated by dividing waist to hip circumference; MRI-derived proton density fat fraction (PDFF) and liver iron corrected T1 (cT1) provided directly by UK Biobank (data-fields 40061 and 40062); liver fat measured by MRS (Dallas Heart Study and NEO studies) or CAP measurement (MAFALDA and Liver BIBLE). Bulk gene expression (RNA-seq) for liver and visceral adipose tissue were derived from MAFALDA study.<br>Whole-genome regression models were adjusted for age, sex, age2, age×sex, age2×sex, the first 10 PCs of ancestry, genotyping array and adiposity index, where adiposity index was VAT, WFM, BMI or no adiposity adjustments. Regression analyses in replication cohorts were adjusted for age, sex, age2, age×sex, age2×sex, the first 10 PCs of ancestry, genotyping array and BMI. Sex-stratified regression models were adjusted for age, the first 10 PCs of ancestry, genotyping array and BMI. In DHS, mean age was XXX years (standard deviation); in MAFALDA, mean age was 44 years (standard deviation 10); in NEO mean age was 56 years (standard deviation 6); and in Liver-BIBLE, mean age was XXX years (standard deviation).
Recruitment	The UK Biobank is a population-based study which has recruited over 500,000 participants aged between 40 and 69 years across the UK between 2006 and 2010, with extensive phenotypic and genetic data. In this study, only individuals of European ancestry were included. Additionally, subjects were excluded if they fall into any of these categories: 1) more than 10 putative 3rd degree relatives, 2) a mismatch between self-reported and genetically inferred sex, 3) putative sex chromosome aneuploidy, 4) heterozygosity and missingness outliers, and 5) withdrawn consent.<br>The NEO study is a population-based study including men and women aged 45 to 65 years, with oversampling of individuals having BMI over 27 kg/m2 from Leiden and surrounding areas in the Netherlands. At baseline, 6,671 participants were included and around 35% of the NEO participants were randomly selected to undergo hepatic triglyceride content (HTGC) measurements by MRS. In the present work, only individuals of European ancestry and available HTGC were included.<br>The Liver-BIBLE-2022 cohort comprises 1,144 healthy middle aged individuals (40-65 years) with metabolic dysfunction (at least three criteria for metabolic syndrome among BMI≥35 Kg/m2, arterial hypertension ≥135/80 mmHg or therapy, fasting glucose ≥100 mg/dl or diabetes, low HDL <45/55 mg/dl in M/F and high triglycerides ≥150 mg/dl) who presented for blood donation from June 2019 to February 2021 at the Transfusion Medicine unit of Fondazione IRCCS Ca’ Granda Hospital (Milan, Italy). Hepatic fat content was estimated non-invasively by controlled attenuation parameter (CAP) with FibroScan® device (Echosens, Paris, France). In the present study, only individuals of European ancestry with genomic data passing quality

control and available CAP measure were included.
The MAFALDA comprises a total of 468 consecutive participants with morbid obesity (BMI ≥35 kg/m2) that underwent bariatric surgery at Campus Bio-Medico University of Rome, Italy in whom SLD diagnosis was assessed by liver histology or vibration-controlled transient elastography including CAP measurement with FibroScan® (Echosens, Paris, France). In the present study, only individuals with Liver fat content estimated by CAP and genotyping available were included.
The DHS is a randomly selected population-based samples study of the Dallas County, Texas, USA. In this study only European Americans individuals from the Dallas Heart Study (DHS-1) where liver triglyceride content was measured by magnetic spectroscopy were included.

Ethics oversight

The UK Biobank received ethical approval from the National Research Ethics Service Committee Northwest Multi-Centre Haydock (reference 16/NW/0274). Data used in this study were obtained under application number 37142. The NEO study was approved by the medical ethical committee of the Leiden University Medical Center (LUMC). The Liver-BIBLE study was approved by the Ethical committee of the Fondazione IRCCS Ca' Granda (ID 1650, revision 23 June 2020). The MAFALDA study has been approved by the Local Research Ethics Committee (no. 16/20) The DHS was approved by the institutional review board of University of Texas Southwestern Medical Centre.

Note that full information on the approval of the study protocol must also be provided in the manuscript.

Field-specific reporting

Please select the one below that is the best fit for your research. If you are not sure, read the appropriate sections before making your selection.

☒ Life sciences ☐ Behavioural & social sciences ☐ Ecological, evolutionary & environmental sciences

For a reference copy of the document with all sections, see nature.com/documents/nr-reporting-summary-flat.pdf

Life sciences study design

All studies must disclose on these points even when the disclosure is negative.

Sample size

The sample size used for multi-adiposity-adjusted GWAS has the largest available number of European individuals for liver fat content (n = 36,394) and liver iron corrected T1 (n=30,481), thus providing adequate power to detect genetic variants of modest effect size. Six of the identified variants from the discovery GWAS were replicated in 4 external cohorts with comparatively smaller sample size (n= 3,903), supporting that our discovery sample size was well-powered to identify moderate effect sizes.

Data exclusions

In the UK Biobank, subjects were excluded if they fall into any of these categories: 1) more than 10 putative 3rd degree relatives, 2) a mismatch between self-reported and genetically inferred sex, 3) putative sex chromosome aneuploidy, 4) heterozygosity and missingness outliers, and 5) withdrawn consent. For all the study cohorts, individuals of self-reported non-European ancestry or with genotype/phenotype unavailable data were excluded. The exclusion criteria were decided a priori.

Replication

The previously unknown genetic loci found in the UK Biobank for liver fat (measured by proton density fat fraction, PDFF) and liver iron corrected T1 were replicated in 4 independent European cohorts: NEO, DHS, MAFALDA and Liver-BIBLE cohorts. The direction of the association in the replication cohorts was consistent with the discovery cohort (UK Biobank) for 6 loci.

Randomization

This is a genetic association study. The study groups are defined based on the genotype and randomized by nature.

Blinding

This was no intervention study thus blinding was not relevant.

Reporting for specific materials, systems and methods

We require information from authors about some types of materials, experimental systems and methods used in many studies. Here, indicate whether each material, system or method listed is relevant to your study. If you are not sure if a list item applies to your research, read the appropriate section before selecting a response.

Materials & experimental systems

n/a	Involved in the study	
☒	☐	Antibodies
☒	☐	Eukaryotic cell lines
☒	☐	Palaeontology and archaeology
☒	☐	Animals and other organisms
☒	☐	Clinical data
☒	☐	Dual use research of concern
☒	☐	Plants

Methods

n/a	Involved in the study	
☒	☐	ChIP-seq
☒	☐	Flow cytometry
☒	☐	MRI-based neuroimaging

Plants

Seed stocks	NA
Novel plant genotypes	NA
Authentication	NA
